# Implementation research to develop and optimize delivery models for evidence-based anemia control interventions in India: Protocol for the precision-driven response for anemia control and sustainable health (PRAKASH) study

**DOI:** 10.1371/journal.pone.0351414

**Published:** 2026-06-18

**Authors:** Priyanka Gupta Bansal, Sarmila Mazumder, Neeraj Sharma, Ragini Kulkarni, Prashanth Thankachan, Pankaj Prasad, Bhuputra Panda, Santosh Kumar Banjara, Bharati Kulkarni, Ranadip Chowdhury, Ketaki Chandiok, Zaozianlungliu Gonmei, on behalf of the PRAKASH Study Group

**Affiliations:** 1 Division of Reproductive, Child Health and Nutrition, Indian Council of Medical Research, New Delhi, India; 2 Society for Applied Studies, New Delhi, India; 3 Department of Operational and Implementation Research, Indian Council of Medical Research - National Institute for Research in Reproductive and Child Health, Mumbai, Maharashtra, India; 4 Division of Nutrition, St. John’s Research Institute, Bangalore, Karnataka, India; 5 Department of Community and Family Medicine, All India Institute of Medical Sciences, Bhopal, Madhya Pradesh, India; 6 School of Public Health, KIIT Deemed to be University, Bhubaneswar, Odisha, India; 7 Indian Council of Medical Research - National Institute of Nutrition, Hyderabad, Telangana, India; AIIMS Jodhpur: All India Institute of Medical Sciences - Jodhpur, INDIA

## Abstract

**Background:**

Government of India launched Anemia Mukt Bharat (AMB) program in 2018. Despite its implementation, the prevalence of anemia remains high. To address the gaps in coverage, adherence and effective implementation, the Indian Council of Medical Research (ICMR) initiated the **Pr**ecision driven **R**esponse for **A**nemia **C**ontrol **a**nd **S**ustainable **H**ealth (PRAKASH) study. It is an implementation research study aimed at co-developing and optimizing a district-level model of evidence-based anemia control interventions. The intended goal is to reduce anemia prevalence to 20% or lower through scalable, context-specific, and sustainable strategies.

**Methods:**

This multi-site study targets six key population groups: children (aged 6–59 months and 5–9 years), adolescents boys and girls (10–19 years), women of reproductive age (20–49 years) and pregnant women. Interventions are anchored in six pillar i. Test–Treat-Track until resolution, ii. prophylactic iron and folic acid supplementation, ensuring high complianceiii. anemia-relevant health interventions, iv. fortified rice distribution, v. dietary diversification and promotion of iron-rich foods, and vi. behavior change communication through Jan Andolan. Anemia prevalence will be estimated through a series of community-based cross-sectional surveys. Mixed methods approach will be employed to assess barriers and enablers at the individual, household, facility, and community levels. The study will utilize implementation science frameworks, including Consolidated Framework for Implementation Research (CFIR) and Expert Recommendations for Implementing Change (ERIC), to guide iterative implementation, real-time model refinement, and monitoring of performance. Information on outcome indicators will be collected to evaluate anemia prevalence, fidelity, feasibility, and service delivery improvements.

**Conclusion:**

The study will try to readdress the implementation challenges across diverse regions and aims to develop a comprehensive and replicable model for the AMB 2.0 program. The study is likely to contribute to the global evidence base on implementation science for anemia reduction in low- and middle-income countries.

## Introduction

Anemia is characterized by low number of red blood cells count or the haemoglobin concentration within them is lower than normal, which impairs the adequate oxygen supply to the tissues and organs [1]. The causes of anemia are multi-faceted comprising of genetic factors, dietary intake, nutritional deficiencies, chronic illnesses, inflammation, infections socio-economic conditions etc. [2–5]. Gender disparity and geographical settings also play significant role in its causation [6,7]. Anemia has several adverse health consequences including impaired cognitive development and stunting in children [8,9], reduced work productivity [10,11], and adverse pregnancy outcomes like stillbirths, preterm birth, low-birth-weight babies, and small for gestational age babies [12–14].

Anemia is a significant public health concern worldwide. According to the World Health Organization’s global anemia estimates, 2025, achieving the 2030 global nutrition target for anemia particularly among women of reproductive age will necessitate accelerated and innovative efforts [15]. India, with its substantial and diverse population, bears one of the highest burdens of anemia globally [16–19]. National surveys such as National Family Health Survey (NFHS)-2–5 have consistently highlighted the high prevalence of anemia, especially among pregnant women, adolescent girls, and young children [20–23]. Despite sustained efforts through programs such as the National Nutritional Anemia Prophylaxis Program and its subsequent expansions, the recent NFHS-5 estimate indicates that 57% of women of reproductive age, 52% of pregnant women, and 67% of under-5 children remain anemic [24]. These data underscore the importance of both the magnitude of the challenge and the opportunity to enhance and accelerate anemia control strategies through evidence-based, innovative, and locally tailored implementation models.

The Government of India launched the Anemia Mukt Bharat (AMB) program in 2018 with a target of reducing anemia prevalence by three percentage points per year across six beneficiary groups: children aged 6–59 months, children 5–9 years, adolescents 10–19 years, women of reproductive age (20–49 years), pregnant women, and lactating women. However, the program faced challenges with implementation variability across states due to limited awareness, side effects affecting compliance, lack of IEC materials, supply chain and logistic gaps, dietary diversity constraints, cultural barriers, behavioural and social factors and the COVID 19 pandemic. To consolidate the scientific foundation of these efforts, on the directives of National Institution for Transforming India (NITI) Aayog, the Indian Council of Medical of Research (ICMR) conducted a comprehensive evidence synthesis and stakeholder consultation exercise. This process reviewed global and national experience, identified best practices, and informed the development of a strengthened package of evidence-based interventions. These recommendations formed the basis of the proposed AMB 2.0, which has been submitted by NITI Aayog to the Ministry of Health and Family Welfare (MoHFW) for consideration. The six interventions—i. Test–Treat-Track until resolution, ii. prophylactic iron and folic acid supplementation, ensuring high complianceiii. anemia-relevant health interventions, iv. fortified rice distribution, v. dietary diversification and promotion of iron-rich foods, and vi. behavior change communication through Jan Andolan. [25–35].

The Precision driven Response for Anemia Control and Sustainable Health (PRAKASH) study has been designed to operationalize and test this evidence-based package through a precision-driven, multi-site implementation research study.The study utilizes a theory of change framework that posits for a precision-driven, district-level model that systematically identifies contextual bottlenecks and co-develops tailored implementation strategies (using CFIR determinants linked to ERIC strategies) that are context-specific, scalable, and sustainable with the aim to improve fidelity, reach, and continuity of the six AMB 2.0 pillars. Importantly, it seeks to address well-recognized programmatic challenges including variable Iron Folic Acid (IFA) coverage and adherence, supply chain bottlenecks, limited access to reliable diagnostic tools, and insufficient integration of strategies to tackle non-nutritional causes of anemia through an implementation science approach. By setting a goal of reducing anemia prevalence to below 20%, PRAKASH study aspires to provide a replicable framework that strengthens anemia control efforts and informs national scale-up.

Through this implementation study we aim to co-develop district model of Anemia Mukt Bharat 2.0 which would be optimized to increase coverage and adherence to reduce anemia prevalence to 20% or lower in beneficiary populations (children 6–59 months, adolescent girls (10–19 years), women of reproductive age (20–49 years), pregnant women). The primary objective would be to co-design, optimize, implement and evaluate a model of implementation strategies to deliver the AMB 2.0 interventions across target beneficiaries and reduce prevalence of anemia to ≤20%. The secondary objectives would focus to achieve high and effective population-based coverage (≥80%) of the AMB 2.0 interventions among target beneficiary groups and document the iterative process and implementation outcomes, in developing the optimized model.

## Methodology

### Study design and setting

The implementation research study will employ a mixed method approach (qualitative and quantitative) for model optimization and interrupted time-series measurements for the outcome evaluation. The six beneficiary groups currently under AMB program: i. Children (6–59 months), ii. Children (5–9 years) iii. Adolescent girls (10–19 years), iv. Adolescent boys (10–19 years) v. women of reproductive age (20–49 years), vi. pregnant women will be included in the study.

Formative research will be undertaken at the individual, community and facility level to identify barriers and facilitators for anemia management implementation across the six selected districts (Table 1). A series of total five cross sectional surveys (consisting of 2 baseline survey, an early implementation, mid implementation and an end-line survey) will be conducted to understand the factors affecting the implementation of the AMB 2.0 program at the individual, facility and community levels and the effectiveness of the implementation program. A thorough evaluation of the healthcare facilities and associated health care workers in the district will also be done to identify gaps that need to be addressed for effective implementation.

**Table 1:** Details of the study districts.

Sites	Anemia Prevalence as per NFHS-5	Geographical Area (Rural to Urban Ratio)
Site 1 Palghar, Maharashtra	Children 6–59 months: 70.3% Women 15–19 years: 52.1% Non pregnant women 15–49 years: 57.2% Pregnant women 15–49 yrs: 48.5%	52%:48%
Site 2 Koppal, Karnataka	Children 6–59 months: 68.1% Women 15–19 years: 45.6% Non pregnant women 15–49 years: 45.7% Pregnant women 15–49 yrs: 43.3%	79%:21%
Site 3 Palwal, Haryana	Children 6–59 months: 71.6% Women 15–19 years: 59.7% Non pregnant women 15–49 years: 57.2% Pregnant women 15–49 yrs: 56.8%	77%:23%
Site 4 Kendujhar, Odisha	Children 6–59 months: 46.6% Women 15–19 years: 68.2% Non pregnant women 15–49 years: 69% Pregnant women 15–49 yrs: 74.7%	86%:14%
Site 5 Sehore, Madhya Pradesh	Children 6–59 months: 82.4% Women 15–19 years: 50.5% Non pregnant women 15–49 years: 44.8% Pregnant women 15–49 yrs: 58.8%	81%:19%
Site 6 Medchal Malkajgiri District., Telangana	Children 6–59 months: 73.8% Women 15–19 years: 56.1% Non pregnant women 15–49 years: 57.4% Pregnant women 15–49 yrs:53.2%	9%:91%

During the implementation phase of the study, the initial model will be co-developed with key stakeholders, drawing on formative research findings and baseline survey results. Model will be refined through iterative, concurrent cycles of implementation, performance monitoring, incorporating stakeholder feedback. At each cycle, emerging barriers and facilitators will be analyzed using implementation science constructs to guide adaptations, thereby progressively optimizing the delivery model. The optimized model with potential to achieve high and effective coverage and impact, will be embedded and expanded across all blocks of the district, with the study team providing structured implementation support to government counterparts to ensure leadership engagement, capacity strengthening, and health system readiness. A predefined set of impact, implementation, and process indicators (as detailed in the later sections) will be monitored. Continuous learning from these indicators, including changes in anemia prevalence, improvements in service coverage, and fidelity to intervention delivery, will generate actionable insights that directly inform refinement of intervention strategies and outreach approaches, enabling adaptive, context-responsive scaling while preserving implementation quality.

### Study sites and participating institutes

The study sites and participating institutes for the PRAKASH study were selected through a transparent and competitive process coordinated by the Division of Reproductive and Child Health Nutrition at ICMR, New Delhi. An Expression of Interest for a “Multistate Implementation Research Study for the Development of a Sustainable, Scalable Model of Implementation Strategies for Prevention and Treatment of Anemia” was published on the ICMR website. A total of 102 proposals were received in response to the call. A screening committee, evaluated proposals based on predefined criteria including study design rigor, prior implementation research experience of the Principal Investigator, engagement with state and district health systems, collaboration with other stakeholders, and potential for integration with national programs. Following this process, six proposals were shortlisted, covering six sites across diverse geographic and socio-cultural settings in India. The participating institutes include ICMR-National Institute For Research in Reproductive and Child Health, Mumbai (Palghar, Maharashtra); St John’s Research Institute, Bangalore (Koppal, Karnataka); Society for Applied Studies, New Delhi (Palwal, Haryana); Kalinga Institute of Industrial Technology, Deemed University, Bhubaneswar and its affiliate institutions – Tata Institute of Social Sciences, Mumbai and InOrder-The Health Systems Institute, Hyderabad (Kendujhar, Odisha);All India Institute Of Medical Sciences Bhopal (Sehore, Madhya Pradesh); and ICMR-National Institute of Nutrition Hyderabad (Medchal Malkajgiri, Telangana). These sites were selected to represent diverse implementation environments and to collaboratively contribute to the development and optimization of a scalable anemia control model.

### Sampling strategy

For sampling purpose, the universe of study would be the entire district. For every cross-sectional survey, four blocks will be covered from the total number of administrative blocks within the district. Village/ward will be the primary sampling unit. Multi stage cluster sampling will be employed. In each selected village/ward, line list of beneficiaries (children, pregnant women, unmarried WRA (where available) and adolescents’ girls) will be collected from ASHA/ANMs/AWWs. It is recognized that the line listing will not have 100% coverage, but as a part of implementation research study, this aspect will be strengthened as interventions are rolled out. Beneficiaries will be selected randomly from the list. For the remaining groups, where ready line listing may not be available, the next 1–2 households where beneficiary was found using line listing, will be used to identify the rest of the beneficiary groups (children 5–9 years; adolescent boys; WRA & adolescent girls (incase CHWs do not have readily available list for WRA) in the stated order of priority. The sample will include a total of 50 beneficiaries from each village/ ward. The method of sampling would be the same in each of the five survey rounds.

**Sample size calculation:** It is assumed that the prevalence of anemia at baseline will be between 40–60% among different age groups as per NFHS-5 & Comprehensive National Nutrition Survey data. Assuming 20% prevalence of anemia at the end-line with 30% relative precision, 95% confidence interval, 1.45 design effect and assuming different non-response rate (40% for children 6–59 months and 5–9 years, 30% for adolescent girls and boys, 20% for women of reproductive age and 20% for pregnant women), the final sample size is calculated as 400 each for children 6–59 months and children 5–9 years, 350 each for adolescent girls and boys, 300 each for women for reproductive age group and pregnant women for each survey rounds. The focus of the study is on all the 6 groups; however, the four groups (children 6–59 months, adolescent girls (10–19 years), women of reproductive age (20–49 years), pregnant women) are selected for the evaluation of impact indicators. For the remaining two groups (children 5–9 y and adolescent boys), the focus is on the process and coverage indicators.

### Ethics approval

The protocol of the study is approved by the Institutional Ethics Committee (IEC) of the participating sites (Ethics Committee for Human Studies, National Institute for Research in Reproductive and Child Health; Institutional Ethics Committee, St. John’s Research Institute; Ethics Review Committee, Society of Applied Studies; Institutional Ethics Committee, Kalinga Institute of Medical Sciences; Institutional Human Ethics Committee, All India Institute of Medical Sciences; Institutional Ethics Committee, National Institute of Nutrition). The study adheres to the principles specified in the Declaration of Helsinki [36]. The study is registered at www.ctri.nic.in (CTRI/2025/06/088025).

Prior to the enrollment, every participant will be provided with a participant information sheet in local language explaining the purpose of the study, its risks, benefits, role of the participants and other details. There will be no coercion of any kind to participate in the study. The participants will not be deprived of any services being offered to them if they decide not to participate in the study. Written informed consent will be obtained from all the participants. For adolescent boys and girls (between 12- < 18 years) written assent will be taken and written informed consent will be taken from the parents/legal guardian. For children, written informed consent will be taken from the parents/legal guardian. The privacy and confidentiality of the participants will be maintained during data collection. The data for the study will be stored in a password protected computer/laptop and only the study investigators and the team members will have access to this data. All the personal identifiers will be removed before publication of results and report submission.

### Comprehensive intervention package: evidence summary

The intervention package tested in the PRAKASH study is grounded in a comprehensive evidence synthesis exercise jointly undertaken by the ICMR, NITI Aayog, MoHFW and partner institutions. This exercise consolidated findings from national program data, research and expert consultations to identify interventions with the strongest potential to reduce anemia in the Indian context. The resulting package, incorporated under AMB 2.0, brings together six pillars: [1] Test–Treat–Track until resolution, [2] prophylactic iron and folic acid supplementation, ensuring high compliance[3] anemia-relevant health interventions, [4] fortified rice distribution through public systems, [5] dietary diversification and promotion of iron-rich foods, and [6] behavior change communication through Jan Andolan. A summary of evidence for the various interventions for anemia reduction has been presented in Table 2.

**Table 2:** Summary of the evidence for various interventions for anemia reduction.

Interventions within AMB	Age-Group	Summary evidence from systematic reviews–
1) Iron Folic Acid Supplementation (for Prophylaxis)	Children under 5 years	RR: 0.61(95% CI 0.50–0.74)^26^
	School-age Children (5–12 years)	RR: 0.50(95% CI 0.39–0.64)^27^
	Women of Reproductive Age	RR: 0.39 (95% CI 0.25–0.60)^28^
	Pregnancy	RR: 0.30 (95% CI 0.19–0.46)^29^
5) Deworming Interventions	Children	RR: 0.82(95% CI 0.60–1.11)^30^
	Pregnant Women	RR: 0.85 (95% CI 0.72–1.00)^31^
	Non-Pregnant Women	MD 3.02 g/L(95% CI 0.1–6.0 g/L)^32^
6) Delayed cord clamping	Early vs. delayed cord clamping in term infants	RR 2.65,(95% CI 1.04 to 6.73)^33^
7) Rice Fortification	General population	RR: 0.72 (95% CI 0.54–0.97)^34^
8) Dietary diversification	Pregnant women	AOR:2.15 (95% CI,1.66–2.65)^35^

RR: Relative Risk; MD: Mean difference; AOR: Adjusted Odds Ratio

These interventions will be implemented through existing health system platforms, Integrated Child Development Services (ICDS), and school health programs, with planning, implementation, and evaluation guided by the Implementation Research Logic Model [37]. Details pertaining to the logic models have been provided in S1 Table.

1. **Test–Treat-Track until resolution**: Systematic annual hemoglobin screening will be conducted for all beneficiaries across the six study sites. This screening will serve as the primary entry point for identifying individuals with anemia. Individuals diagnosed with anemia will receive appropriate treatment and be monitored until resolution. Mechanisms will be in place to track both nutritional and non-nutritional causes of anemia, such as sickle cell disease and thalassemia. The strategy will primarily focus on four high-burden groups children 6–59 months, adolescent girls, pregnant women, and women of reproductive age. Adolescents’ boys and younger children (5–9 years) will also be included in the strategy. Regular quarterly follow-ups by frontline workers will help ensure treatment compliance, early relapse detection, and sustained recovery.
2. **Prophylactic iron and folic acid supplementation:** In parallel with screening, prophylactic IFA supplementation will be implemented to mitigate the risk of anemia across populations. ASHAs and Anganwadi workers will maintain updated beneficiary line lists and ensure the distribution of tablets or syrups in accordance with program guidelines. Counseling and follow-up will be emphasized to enhance adherence, address common barriers such as side effects, and ensure continuity of prophylaxis.
3. **Anemia relevant health interventions:** To comprehensively address anemia, the intervention package will integrate maternal, child health, and infection-control practices. This includes promotion of delayed cord clamping at birth, iron supplementation for low-birth-weight infants, and routine deworming. Water, Sanitation, and Hygiene (WaSH) behaviors will be promoted as preventive measures. Additionally, individuals diagnosed with acute or chronic infections such as malaria, tuberculosis, HIV, or hepatitis will also undergo anemia screening and receive appropriate management, thus linking infectious disease control with anemia reduction.
4. **Fortified rice through Public Distribution Systems:** Nutrient security will be strengthened by leveraging government-supported food distribution programs. Fortified rice is already being distributed through the Public Distribution System (PDS), Integrated Child Development Scheme (ICDS), and Pradhan Mantri Poshan Shakti Nirman (PM-POSHAN) (mid-day meals) but through the study there will be efforts to generate community awareness and sensitization campaigns to promote acceptance and regular consumption of fortified rice. Monitoring will be conducted using official distribution records and household surveys to assess uptake and effectiveness in addressing dietary iron gaps.
5. **Dietary diversification and promotion of iron-rich foods:** Dietary modification is an essential pillar for long-term anemia prevention. The intervention will promote consumption of iron, folate, and vitamin B12-rich foods, including millets and other traditional staples. Beneficiaries will be counseled at Village Health and Nutrition Days, Ayushman Arogya Mandirs, Self-Help Groups, and Anganwadi Centres. Practical demonstrations such as recipe sessions and use of dietary diversity checklists will reinforce key messages. Schools will serve as a critical platform, incorporating nutrition education into the curriculum, training student health ambassadors, and organizing activities under the “Eat Right” campaign. Program menus under PM-POSHAN and ICDS will also be reviewed to align with micronutrient standards. Communication strategies will employ Information, Education, and Communication (IEC) materials, Short Message Service, social media, and Ayush Ahaar campaigns to reinforce consistent dietary practices.
6. **Behaviour change communication through Jan Andolan:** Finally, community engagement through Jan Andolan will ensure sustainability and collective responsibility for anemia control. This approach will emphasize advocacy, capacity building, and partnerships with schools, self-help groups, and local organizations. Social mobilization efforts will highlight the importance of annual screening, adherence to IFA supplementation, acceptance of fortified foods, and inclusion of diverse diets in daily meals. By positioning anemia reduction as a community priority, the intervention aims to build momentum toward a sustained, population-level movement.

Together, these six strategies create a comprehensive and integrated package that combines biomedical, nutritional, and behavioral approaches. By placing annual anemia screening at the center, the PRAKASH study seeks to ensure early identification and continuous management, while reinforcing prevention through supplementation, food-based strategies, fortified staples, and community mobilization. This integrated package is expected to provide a scalable and sustainable model for anemia prevention and control.

A detailed overview of the delivery mechanism of the 6 intervention strategies has been provided in S2 Table.

### Conceptual framework

**Fig 1:**
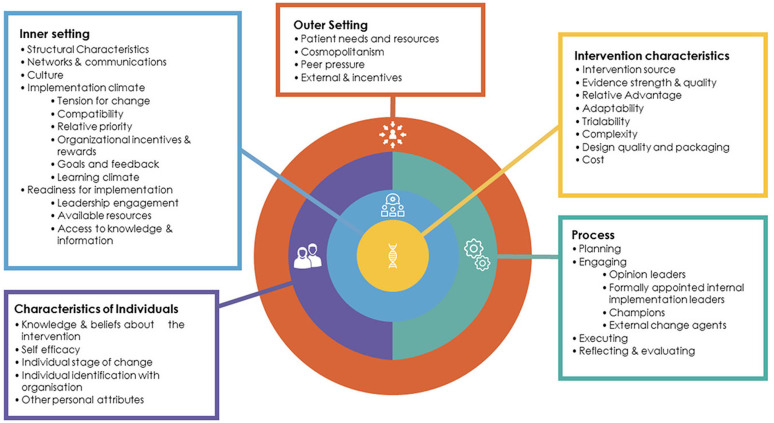
CFIR framework highlighting the five domains along with the underlying constructs.

The PRAKASH study will be guided by the Consolidated Framework for Implementation Research (CFIR) and the Expert Recommendations for Implementing Change (ERIC). CFIR provides a structured lens to examine contextual determinants of implementation across five domains, while ERIC offers a menu of evidence-informed strategies to address identified barriers. Together, these frameworks will inform the design and delivery of the intervention package, guide model development, and define indicators for monitoring performance. A detailed list of the five domains along with the underlying constructs has been provided in Fig 1 [38]. Based on the initial formative research, stakeholder inputs, and field experience, a prototype set of strategies has been identified for the CFIR constructs and will be refined iteratively during implementation.

1. **Innovation (intervention) characteristics:** A central construct is adaptability, ensuring that the evidence-based AMB 2.0 interventions are responsive to local needs and preferences. Local needs assessments and community engagement will help tailor service delivery strategies. For example, if supplementary nutrition programs are perceived as poor quality or underutilized, discussions will be held with service providers, supervisors, and program managers to adapt delivery mechanisms.
2. **Outer setting:** This domain reflects the wider policy, socio-economic, and cultural environment. Two key constructs are cosmopolitanism and patient needs and resources. Strategies include building coalitions with state and district health authorities to ensure alignment with existing programs, mobilizing communities through social and behavior change communication (SBCC), and increasing demand for anemia services. Awareness campaigns will use a mix of mass media (print, audiovisual, social media) and community-level platforms such as street theatre, school programs, and health fairs to reinforce the importance of anemia prevention and management.
3. **Inner setting:** The organizational environment in which interventions will be delivered include health facilities, schools, and ICDS centers. Structural gaps will be addressed through strategies such as improving physical infrastructure, strengthening record systems for anemia testing and tracking, and decentralizing service delivery points where access barriers exist. Readiness for implementation will be enhanced through leadership engagement and technical support units comprising ICMR experts, state authorities, and local partners. These units will provide ongoing guidance, periodic review, and facilitate partnerships with academic institutions for training and capacity building. Hands-on training and supportive supervision for frontline workers will focus on practical counseling and problem-solving skills.
4. **Characteristics of individuals:** Successful implementation will also depend on the motivation, knowledge, and capacity of individuals engaged in delivering or receiving interventions. These include beneficiaries (children, adolescents, women), community health workers, nurses, doctors, ICDS supervisors, school personnel, and local administrators. Tailored training, role clarification, and supportive supervision will strengthen individual commitment and capacity to deliver interventions effectively.
5. **Implementation process:** This domain emphasizes stakeholder engagement, execution, reflection, and evaluation. Strategies include conducting educational meetings and outreach visits to build knowledge, developing an implementation blueprint with clear goals, milestones, and indicators, and preparing user-friendly educational and communication materials. Local consensus discussions will foster shared ownership, while champions and opinion leaders will be identified to advocate for anemia control. Iterative cycles of implementation will involve small tests of change, concurrent monitoring, and feedback to refine strategies. Tools for quality monitoring, periodic audits, and readiness assessments will ensure that barriers and facilitators are continuously identified and addressed.

By applying CFIR to identify determinants and ERIC to operationalize strategies, the PRAKASH study aims to create a dynamic, context-sensitive, and scalable model for implementing evidence-based interventions to reduce anemia.

### Research teams

The research team will support the government in implementing the interventions assisting the government in resolving the barriers, providing feedback and inputs on performance and optimization processes during co-design workshops, and collecting research data on predefined outcome indicators.

**Central coordinating team (CCT)**: The team will plan and strategize the research activities, ensures the strategic execution in the respective sites. It will also lead the protocol and tool development, establish a relationship with all study sites at district and state level as required, lead the co-development of strategy and implementation plan based on learning from formative research and participatory planning. Quality assurance visits will also be undertaken by the team for monitoring and evaluation of project activities, manage and analyze the data and develop report.

In each study site, the following teams will operate at different levels to orchestrate the conduct of the study which will comprise of:

**Program learning team (PLT):** The team will conduct formative research for implementing the AMB interventions to inform the development of the initial model, undertake continued program learning activities to identify barriers, facilitators and conduct root cause analysis using qualitative research methods. Information on process evaluation indicators will also be collected by the PLT.

**Implementation support team (IST):** The team will handhold the government partners, demonstrate the pragmatic steps of implementation, navigate the barriers, and leverage on the facilitators effectively at facility levels and in the community. This would entail complete and valid documentation of services in the registers/electronic data system to ensure periodic extraction and review of the data. The IST will also facilitate preparedness of the facilities for infrastructure, human resource, training, logistic arrangement including job aid, communication material

**Outcome monitoring team (OMT):** An independent group of researchers will collect quantitative data and monitor the predefined primary and secondary outcomes indicators throughout the implementation phase. Information on the cross-sectional surveys will also be collected by the OMT.

### Study phases

**Fig 2:**
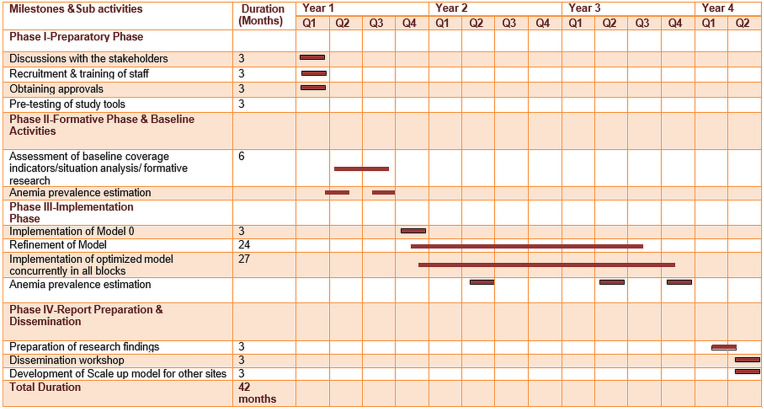
Gantt chart specifying the study timelines.

The PRAKASH study will be conducted over a period of 42 months in four sequential phases, each with specific objectives and activities. A gantt chart specifying the timelines of the study phases are presented in Fig 2.

**Phase I: Preparatory phase (0–3 months)**-The preparatory phase will focus on establishing the groundwork for study implementation. Activities will include stakeholder consultations at state, district, and local levels; hiring and training of study teams; obtaining regulatory and ethical approvals; and pre-testing of data collection tools to ensure contextual relevance and feasibility.

**Phase II: Formative phase and baseline activities (4–9 months)**- This phase will generate evidence on the existing status of anemia management and implementation gaps in the AMB program. Two baseline surveys will be conducted at months 3 and 9 to establish anemia prevalence across six priority beneficiary groups (children 6–59 months and 5–9 years, adolescent girls and boys, pregnant women, and women of reproductive age). Mixed-methods research will assess system-level barriers and facilitators, including service delivery quality, provider knowledge and skills, beneficiary awareness and compliance, procurement systems, and laboratory support. In addition, the formative phase will review the status of AMB interventions such as delayed cord clamping, deworming, fortified rice distribution through social welfare schemes, and SBCC activities related to dietary diversity and anemia prevention.

**Phase III: Implementation phase (10–39 months)**- The implementation phase will operationalize and refine a district-specific intervention model using an iterative and adaptive approach. An initial “Model 0” will be developed through co-design workshops with stakeholders, based on formative research findings. This model will be piloted, refined, and optimized (Models 1, 2, etc.) through continuous cycles of implementation, monitoring, and feedback. Special strategies will be included to reach non-school-going children and women of reproductive age. Cross-sectional surveys will be conducted at months 18, 30 (early implementation and mid implementation survey) and 36 (an end-line survey) to monitor outcome indicators, including anemia prevalence. Process indicators will be tracked through health facilities and sub-centers, complemented by qualitative assessments such as observations, in-depth interviews, and focus group discussions. Data from multiple sources will be triangulated to identify persistent barriers and enablers, with findings continuously fed back to stakeholders for course correction. The optimized implementation model will be concurrently applied across all blocks of the study district with support to government personnel.

**Phase IV: Report preparation and dissemination (40–42 months)**-The final phase will focus on synthesis and dissemination of study findings. Activities will include preparation of comprehensive reports, stakeholder dissemination workshops at district, state, and national levels, and development of a scale-up model adaptable to other sites with necessary contextual modifications.

### Outcome assessment

The outcome monitoring team will conduct five surveys throughout the study. The key indicators to be assessed are categorized in two broad categories:

**1. Impact level Indicator:**
  - **Primary outcome** will include prevalence of anemia among children 6–59months, adolescent girls (10–19yrs), and women of reproductive age and pregnant women (all trimesters)
  - **Secondary outcomes** would include [1] prevalence of moderate anemia among children 6–59 months, adolescent girls (10–19 yrs), women of reproductive age and pregnant women (all trimesters); [2] prevalence of severe anemia among children 6–59 months, adolescent girls (10–19 yrs), women of reproductive age and pregnant women (all trimesters)
**2. Implementation and Coverage Indicators:**

The implementation strategies covering all 6 domains of interventions will impact on services and coverage outcomes which will be captured as secondary outcomes. The implementation indicators (intervention-wise wise) will include:

### Test, treat, and track individuals with anemia

- Proportion of women and children
  i. tested for anemia (by group)
  ii. anemic (mild, moderate, severe) (by group)
- Proportion of anemic individuals
  i. initiated on treatment
  ii. taking treatment for >75% days
  iii. who consumed: at least 75% of prescribed dose, 50–75% dose, less than 50% dose
  iv. with Hb tested every 3 months until resolution of Anemia
  v. who improved (or recovered) after 3 months of treatment
  vi. who did not improve & needed additional tests, received additional testing
  vii. who were referred to a specialist received a consultation& further treatment

### Prophylactic iron and folic acid supplementation

Proportion of women and children given prophylactic supplements who consumed >75% doses

### Anemia-relevant health interventions

Proportion of

i. babies received delayed cord clamping
ii. LBW infants given prophylactic Iron supplements who consumed>75% doses
iii. Proportion of eligible women and children who received de-worming doses
iv. LBW and Preterm babies screened for anemia
v. TB, HIV, malaria patients screened for anemia.

**Eat right to prevent anemia:** Proportion of women and children consumed iron, folic acid, Vitamin B12 rich foods.

**Consumption of fortified rice:** Proportion of households consumed fortified rice.

**Jan Andolan for anemia control through behaviour change:**

i. Proportion of beneficiaries involved in community discussions on anemia
ii. Dietary practices (consumption frequency of iron rich foods)
iii. Awareness levels for anemia control among the beneficiaries
**3. Process Indicators**

The Implementation Support Team (IST) will focus on quantitative data collection, utilizing sources such as Health Management Information System (HMIS), health facility records, records of community health workers, Anganwadi workers and schools/Rashtriya Bal Swasthya Karyakram/Rashtriya Kishore Swasthya Karyakram etc. A detailed list of the indicators to be collected by the IST has been provided in Supplementary Table 3. In parallel, the Program Learning Team (PLT) will gather qualitative insights to investigate the reasons for compliance or non-compliance (for >75% dose, anemia testing among those with chronic/acute infection, delayed cord clamping, to management algorithms), inability to screening, conducting or not conducting SBCC and reasons for use/non- use of IEC materials developed by the responsible stakeholders, underlying reasons for good/poor adherence to AMB algorithms as recommended simultaneously providing with recommendations and valuable feedback to refine outreach efforts.

The PLT will also provide updates on the percentage of sessions observed to assess whether anemia diagnostic tools are used as recommended. The percentage of stakeholders demonstrating adequate knowledge, positive attitudes and appropriate practices will also be collected by the PLT.

Information on implementation- and process-specific indicators such as adherence to defined intervention protocols, documentation of iterative adaptations undertaken during the course of implementation, and systematic recording of key delivery processes would also be captured.

### Data quality and management

Data will be collected in electronic data capture forms (eCRFs) through the REDCap online software. The eCRFs will have an in-built range and logical checks. Data quality will be frequently checked at two levels: at the site by data management team led by site investigators and centrally by the central implementation team (at ICMR).

To ensure study quality,10% of the collected data will be rechecked by the site PI/coordinator and/or the Central Coordinating Unit. Multilevel quality assurance measures will be performed to ensure the correctness, completeness, and timeliness of the data. Each team will ensure the implementation with rigor. They will conduct regular oversight and monitoring of all study activities through regular field visits with joint participation of the Implementation team members. The management and review of the database will be carried out by the team coordinators responsible for their respective areas. The data entry system will incorporate built-in mechanisms for performing range and consistency checks.

### Data analysis

**Quantitative data:** Bivariate analyses will be conducted using chi-square tests for categorical variables and t-tests or non-parametric equivalents for continuous variables to assess associations between outcomes and potential time-varying confounders. To account for clustered data (e.g., individuals nested within facilities and blocks) and potential intra-cluster correlation, analyses will incorporate cluster-adjusted methods. Implementation indicators, including testing, treatment, and tracking of anemia cases; prophylactic iron and folic acid supplementation; anemia-related health interventions; dietary practices (Eat Right initiatives); consumption of fortified rice; and community-based initiatives (Jan Andolan for anemia control) will be summarized using proportions and assessed over time. Pre–post intervention comparisons will be conducted using McNemar’s test for paired categorical outcomes and paired t-test or Wilcoxon signed-rank test for continuous outcomes. Trends across time will be evaluated to compare pre- and post-intervention phases. A p-value < 0.05 will be considered statistically significant. Time series plots will be examined to visualize underlying trends, seasonal patterns, and potential outliers. Multilevel interrupted time series (segmented regression) or mixed-effects regression models will be used as appropriate. SPSS software version 22 will be used for analyzing the quantitative data collected [39]

**Qualitative data:** The interviews will be audio-recorded. Transcriptions will be prepared from the audio recordings and field notes. The NVivo software package will be used to analyze the data, which will be done simultaneously with data collection. Responses will be coded. The codes on the same theme will be grouped into themes and sub-themes. The findings will be weighted by identifying the key themes and estimating the number of times the theme appears and the number of respondents who mentioned the theme. A framework analysis will be used, primarily a case and theme-based analysis. A matrix will display the information and enable us to examine information across rows, which will help maintain the context, and down the columns, which will help in theme development. The collected data will be analyzed through NVivo version 15 software [40].

### Dissemination strategy

The findings of the implementation research will be disseminated at various levels. At the community and implementation level, details pertaining to intervention coverage, acceptability, and effectiveness will be shared through community meetings, frontline worker debriefings, and district-level review platforms to support real-time feedback and local adaptation of strategies.

At the program and policy level, interim and final results on coverage, anemia reduction trends, and key implementation outcomes (e.g., feasibility, fidelity, and scalability) will be disseminated to state and national health authorities through policy briefs, technical reports, and stakeholder workshops. These outputs will directly inform state action plans and support scale-up of the optimized AMB 2.0 model.

At the scientific and professional level, the effectiveness of the intervention model, time-series evaluation results, and lessons from the iterative implementation process will be disseminated through peer-reviewed open-access publications and presentations at national and international conferences. Additionally, implementation toolkits and operational guidelines derived from the co-designed model will be developed and shared with program managers and implementing agencies to facilitate replication and scale-up in similar settings.

## Discussion

This study aims to reduce the prevalence of anemia through a comprehensive, multi-level intervention package that incorporates the intervention strategies as outlined in the AMB program. Implementation of health initiatives like the AMB program often experiences challenges at the individual, household, facility, and community levels. These include limited awareness, misconceptions, and cultural taboos (surrounding anemia, its symptoms, and treatment modalities) among beneficiaries and their families, community perceptions, inadequate training and knowledge among the service providers, attitudinal and workload barriers, data maintenance and monitoring gaps, operational dynamics of healthcare organizations as well as systemic coordination gaps. To address these multifaceted challenges, the study employs a mixed-methods approach within the CFIR-ERIC strategies framework to assist in the identification of factors that facilitate and impede implementation, while concurrently suggesting strategies that would help in streamlining a smoother implementation to bridge implementation gap and improve reproducibility as well as transparency to ensure maximum public health impact.

Implementation research is known to be crucial for addressing challenges by investigating the practical aspects of program execution and developing strategies to enhance effectiveness in real life settings. Implementation research can systematically study challenges, offer evidence-based solutions, and optimize strategies for scaling up interventions. In the context of this, the findings from the present study are likely to offer valuable implementation-level insights that can inform scale-up efforts for the AMB 2.0 program. The learnings from the study are intended to inform and guide the implementation of the program and provide empirical evidence on feasibility, acceptability, and operational considerations across real-world settings. Integrating these learnings into the design and delivery of AMB 2.0 may facilitate a more systematic, context-responsive, and effective scale-up, thereby enhancing the program’s potential for sustained population-level impact. Recent studies emphasize the importance of understanding and overcoming barriers in health program implementation. Evidence shows that context-specific adaptations and stakeholder engagement are vital for improving program outcomes [41–43]. Additionally, continuous quality improvement approaches have proven effective in health programs by addressing process inefficiencies and enhancing service delivery [44]. The concentrated efforts through this study are bound to be of significance not only in reducing the prevalence of anemia across the selected six geographically and culturally diverse populations, but also ensuring increased intervention coverage, utilization of the health care services while concurrently strengthening the health care ecosystem. This study is also expected to be of immense utility to the policy makers and public health workers in diverse regions of the country as well as other low- and middle- income countries and will ensure a greater scalability of the study in developing concentrated efforts that are needed to alleviate the persistent issue of anemia in women, children and the adolescent population, driving a new India-specific precision delivery model for AMB 2.0 optimization. The findings are likely to inform the rollout of the AMB 2.0 by the Government of India, with key learnings supporting implementation across districts nationwide. Furthermore, the insights generated may be adaptable to similar health system contexts in other LMICs, enhancing the broader relevance and applicability of the study.

## Supporting information

S1 Table Theory Logic Model Framework to be implemented under the PRAKASH study. The table demonstrates the component (inputs, activities, outputs, outcomes and impact) wise framework of the 6 intervention strategies.
(DOCX)

S2 Table Platforms, delivery mechanisms, and implementation activities for intervention delivery. The tables present the platforms and mechanisms used to deliver interventions across beneficiary groups under the Test, Treat and Track strategy, prophylactic iron and folic acid (IFA) supplementation, and de-worming, as well as key activities undertaken during the model optimization and scale-up phases.
(DOCX)

S3 Table Indicators to be collected by IST-From HMIS and other source The table demonstrates the indicators that will be collected from the HMIS and other sources. (DOCX)
