# Supplementary material for: Implementation research to develop and optimize delivery models for evidence-based anemia control interventions in India: Protocol for the precision-driven response for anemia control and sustainable health (PRAKASH) study
Source: PLoS One. 2026 Jun 18;21(6):e0351414. doi: 10.1371/journal.pone.0351414 (PMC13278398; doi:10.1371/journal.pone.0351414)
Supplement: S1 Table — The table demonstrates the component (inputs, activities, outputs, outcomes and impact) wise framework of the 6 intervention strategies. (DOCX) [file pone.0351414.s001.docx]

| **Intervention** | **Inputs** | **Activities** | **Outputs** | **Outcomes** | **Impact** |
| --- | --- | --- | --- | --- | --- |
| 1. Test, treat, and track individuals with anemia | 1. Availability of diagnostic tools at health facilities/ other testing platforms like school, AWCs 2. Trained healthcare personnel(e.g., ASHAs, ANMs) 3. Existing treatment protocols and supplements 4. Establishment of tracking system, preferably electronic, if not,register and phone based tracking. 5. Dedicated day for non- pregnant WRA to visit health facility (govt consensus) | 1. Annual anemia screening at required frequency of vulnerable groups at decided platforms 2. Initiation of treatment and counseling f or those with anemia 3. Follow-up visits by community health workers (eg. ASHA/ANM) to check compliance to treatment & reinforce importance of IFA 4. Tracking of those with anemia by CHO/ANM/MO, repeat Hb and appropriate treatment as per algorithm; follow up till resolution of anemia | 1. %women, adolescent girls and children tested for anemia (by group) 2. %anemic individuals initiated on treatment 3. %anemic individuals taking treatment >75% days 4. % anemic individuals who consumed: at least 75% of prescribeddose,50-75%dose, less than 50% dose 5. %anemic individuals with Hb tested every 3 months until resolution of anemia 6. %those who did not improve& needed additional tests, received additional testing 7. %those who were referred to a specialist received a   Consultation & further treatment | 1. % anemic individuals whose anemia resolved within 9 months of diagnosis | 1. Prevalence of anemia (and moderate or severe anemia) in a random sample of women and children |
| 2.Prophylactic Iron and Folic Acid supplementation | 1. Adequate stock of IFA 2. Knowledge and attitude of Healthcare workers (e.g., ASHAs, ANMs   &Teachers) about dose, regime and frequency fordifferent beneficiary groups | 1. Distribution of IFA supplements through healthcare workers, Teachers etc. 2. Regular follow-upto ensure compliance 3. Creating awareness in the community to ensure higher compliance | 1. % of non-anemic women, adolescent girls, and children given prophylactic IFA supplements 2. % of women and children aware of the importanceof anemia and prophylactic supplements | 1. % women and children given prophylactic supplements who consumed >75% doses | 1. Prevalence of anemia (and moderate or severe anemia)in a random sample of women and children who were non-anemic |
| 3. Anemia relevant health interventions | 1. Health care staff trained in anemia relevant health interventions 2. Anthelmintic drugs available at health centres/schools | 1. Distributing deworming tablets through platforms like National Deworming Days (NDD)/ Schools/ AWCs etc. 2. Incorporating anemia relevant health interventions | 1. %birth attendants who report delayed cord clamping 2. %of health workers who report testing and treating for anemia for patients diagnosed with chronic infections | 1. % of eligible women, adolescent girls and children who received de worming doses | 1. Prevalence of anemia (and moderate or severe anemia)in a random sample of women   and children |

Supplementary Table 1 Theory Logic Model Framework to be implemented under the PRAKASH study

| **Intervention** | **Inputs** | **Activities** | **Outputs** | **Outcomes** | **Impact** |
| --- | --- | --- | --- | --- | --- |
|  |  | In all relevant programs | 1. Increase in distribution of deworming tablets |  |  |
| 4. High coverage of fortified rice in public distribution systems | 1. Availability of Fortified rice through PDS/ICDS/other welfare schemes | 1. Distribution of fortified rice using current PDS, MDM, ICDS infrastructure 2. Utilize existing community channels to promote the benefits of fortified rice | 1. Quantity of fortified rice distributed through existing channels 2. Awareness among community of benefits of fortified rice consumption | 1. % of households consumed fortified rice | 1. Prevalence of anemia (and moderate or severe anemia)in a random sample of women and children |
| 5.Eat right to prevent anemia | 1. BCC/educational materials to promote Eat Right for Anemia 2. Training of healthcare workers about iron rich foods | 1. Conducting nutrition counselling at various platforms like VHND,AWC etc. 2. Activities like Iron rich recipe demonstration during Poshan Maah 3. Emphasis on iron rich diet to address anemia using schools as a platform | 1. Number of counselling sessions conducted 2. Increased knowledge of community/school children on iron, FA, Vit B12 rich foods | 1. % of women and children consumed iron, folic acid, Vitamin B12 rich foods | 1. Prevalence of anemia (and moderate or severe anemia)in a random sample of women and children |
| 6. JAN ANDOLAN for  anemia control through behaviour change | 1. IEC and BCC materials 2. Network of community health volunteers/ celebrities/influencers 3. Availability of Media platforms like social media, mass media etc. | 1. Organize campaigns for anemia awareness 2. Engage communities through health activities & programs 3. Disseminate information using existing media channels | 1. Reach and engagement using current campaigns 2. Improved awareness and attitudes towards anemia prevention and treatment | 1. % of beneficiaries involved in community discussions on anemia | 1. Prevalence of anemia (and moderate or severe anemia)in a random sample of women and children |
