# Supplementary material for: Implementation research to develop and optimize delivery models for evidence-based anemia control interventions in India: Protocol for the precision-driven response for anemia control and sustainable health (PRAKASH) study
Source: PLoS One. 2026 Jun 18;21(6):e0351414. doi: 10.1371/journal.pone.0351414 (PMC13278398; doi:10.1371/journal.pone.0351414)
Supplement: S2 Table — The tables present the platforms and mechanisms used to deliver interventions across beneficiary groups under the Test, Treat and Track strategy, prophylactic iron and folic acid (IFA) supplementation, and de-worming, as well as key activities undertaken during the model optimization and scale-up phases. (DOCX) [file pone.0351414.s002.docx]

**Supplementary Table 2a : Platform and mechanisms for delivering interventions under Test, Treat and Track strategy**

| **Beneficiary Groups** | **Test. Treat and Track** | | | | | | |  |
| --- | --- | --- | --- | --- | --- | --- | --- | --- |
|  | **Test** | | **Treat** | | **Track** | | |  |
|  | **Platform** | **By whom and**  **how** | **Platform** | **By whom** | **Platform** | **By whom** | |  |
| **Children(6-59 months)** | All children(<3yrs) to be screened once a year during Immunization visits. Additionally, for clinically visible anemic cases, Point of Care (POC) will be done*  Children >3 yrs- Testing will be done annually at the AWC by Rashtriya Bal Swasthya Karyakram (RBSK)  Mobile Health teams or at camps organized by Govt. health depts.  Further, POC if clinically visible anemia even before 1 year; annually is too long a gap; this will be discussed with the government and implemented based on consensus. HWs will be trained in identification of clinically visible anemia. | Auxiliary Nurse Midwife (ANM)/Staff nurse/ Medical Officer (MO)  RBSK Mobile Health teams  All Accredited Social Health Activist(ASHAs) and Anganwadi Worker (AWWs) will maintain a line list of all children in this age group in the area covered by them.  Besides digital hemoglobinometers, use of Non invasive Hb meter/ Minimally invasive POCT will also be explored | First bottle at Immunization platform for children who are due for immunization. For those children who are not due or do not come for immunization, the Iron Folic Acid (IFA) will be given by RBSK/ Health & Wellness Centres (HWC)/ Primary Health Centers (PHC)/ Community Health Centers (CHC)  Subsequently from AAM.  Children>3yrs- Ayushman Arogya Mandir (AAM) | ASHA will follow up fortnightly.  Community Health Officer(CHO) in AAM  and AWWs will also be involved in dispensing the IFA.  Overall responsibility: ANM | Tracking of those with anemia, repeat Hb and appropriate treatment till resolution of anemia as per the algorithm.  The ASHAs can be trained in POC and given the device for repeat Hb tests if the family refuses or cannot come to the facility or AAM.  The IST will explore the option of developing an App based tracking system, either developed by the IT within the health systems or IT specialist hired by the government. Till such as App is developed, registers will be maintained with appropriate rows and columns and complete identity of each child with anemia, with follow up dates entered, reminders fed into the mobile phones. | ASHA/ CHO/ ANW/  MO and if needed AWW | |  |
| **Children(5-9 years)** | All children to be screened once a year. Additionally, for clinically visible anemic cases, POC will be done. School Going- In schools; camps organized by Govt. Health dept. Out of School or going to private schools that do not have screening programs- AAM, Poshan Maah, Rashtriya Kishore Swasthya Karyakram (RKSK), Other health camps/ AMB Camps | In School: RBSK team; Government Health Department or private  Health facilities  Out of School: CHO  RKSK  All ASHAs and AWWs will maintain a linelist of all children in this age group in the area covered by them. | Govt. Schools: In school distribution Private Schools: From AAM through cluster resource centre Out of School: IFA will be procured from AAM | In School: Class Teachers  Out of school children: Home visits by ASHA every month  ASHAs, ANMs  will additionally check coverage based on their line list of beneficiaries in these age groups and confirm whether screening and treatment were provided at school/by RKSK  or AAM. | Tracking of those with anemia, repeat Hb and appropriate treatment till resolution of anemia as per the algorithm.  Tracking Same as above | CHO/ANM  /MO/ ASHA  MO of RKSK | |  |
| **Adolescents (10-19years)** |  |  |  |  |  |  |  |  |
| **WRA(20-49**  **years)** | Annual Screening Village Health and Nutrition Day **(**VHND)/ Urban Health and Nutrition Day (UHND)/AAM  During annual Non Communicable Diseases (NCD) Screening  A particular day of the month (like Pradhan Mantri Surakshit Matritva Abhiyan (PMSMA))/  designated day at PHC or AAM/Family Planning | 20-30Years:ANM  30-49years:CHO along with NCD screening  All ASHAs and AWWs will maintain a line list of all WRA in the area covered by them, they already maintain the eligible couple  register. | 20-30 Years: At VHND/UHNDby ANM.  Subsequently from AAM  30-49years:At AAM by CHO  Along with NCD screening  Urban areas with no ASHA: at testing platform (health camp etc.) | Home visits by ASHA every month | Tracking of those with anemia, repeat Hb and appropriate treatmenttill resolution of anemia as per algorithm.  Tracking same as above | | CHO/ANM  /MO AWWs | |
| **Pregnant women** | At every ANC (at least 4 times) ANC/VHND/  AAM, PMSMA | ANM  All ASHAs and AWWs will maintain a line list of all pregnant women in the area covered by them. | Ante Natal Care (ANC) clinic/VHND/ PMSMA/UHND | Home visits by ASHA every month | Tracking of those with anemia, repeat Hb and appropriate treatment till resolution of anemia as per the algorithm  In addition to the 4 ANC visits, the same tracking system will be applicable | | CHO/ANM  /MO  ASHAs/A WW | |

*****MOs/staff nurses/ANMs/CHOs/RBSK team will be trained to identify signs of anemia. It will be ensured that all children in this age group in the area covered by each ASHA/AWW, are examined by any one of them once in 3 months to identify anemia in this age group

**Supplementary Table 2b: Platform and mechanisms for delivering interventions under Prophylactic IFA supplementation**

| **Beneficiary** | **Platform** | **By whom** |
| --- | --- | --- |
| **Children (6-59 months)** | Distribution during VHND/UHND/ Vit A round/Home visits/Immunization facility/AWC | ASHA/AWW |
| **Children (5-9 years)** | School-going children: In school on a fixed day, once a week Out of school children: IFA tablets will be distributed by ASHA at home | School teachers ASHA CHO RBSK AWW |
| **Adolescents (10-19 years)** | School-going children: In school on a fixed day, once a week Out of school children: IFA tablets will be distributed by ASHA at quarterly Adolescent Health Day component of RKSK programme at AWCs | ASHA CHO RKSK |
| **WRA (20-49 years)** | Women (20-30 yrs)- Distribution during VHND >30 years- AAM during annual NCD screening | ASHA/CHO MO |
| **Pregnant women** | IFA tablets will be distributed during ANC contacts | ASHA/ANM |

**Supplementary Table 2c: Platform and mechanisms for delivering intervention under De-worming**

| **Beneficiaries** | **Dose and regime** | **Platform** |
| --- | --- | --- |
| **Children 12–59 months** | Biannual dose of 400 mg albendazole (1⁄2 tablet to children 12–24 months and 1 tablet to children 24–59 months) | Anganwadi centres by AWWs, ASHAs RBSK |
| **Out of school children and adolescents (1-19 yrs)** | Biannual dose of 400 mg albendazole (1 tablet) on designated dates | Anganwadi centres by AWWs, ASHAs RBSK, RKSK |
| **School- going children and adolescents (1-19 yrs)** | Biannual dose of 400 mg albendazole (1 tablet) on designated dates | School ASHAs RBSK, RKSK |
| **Women of reproductive age (non-pregnant, nonlactating) 20– 49 years** | Biannual dose of 400 mg albendazole (1 tablet) | National Deworming Day /Dedicated day for Womem of Reproductive Age based on Government consensus |
| **Pregnant women** | One dose of 400 mg albendazole (1 tablet), after the first trimester, preferably during the second trimester | ANC clinics/ VHND/ PMSMA |

**Supplementary Table 2d: Key activities to be conducted during the model optimization and scale up phases**

| **Strategies to Identify Beneficiary Groups** | Community Mapping and Surveys Household Visits Mapping of Schools and AWCs Data from health systems Facility Mapping |
| --- | --- |
| **Strategies to Improve Coverage of Beneficiary Groups for Prophylaxis and Treatment- increased service utilization** | Setting up of mobile health clinics and camps for anemia screening  Targeted awareness campaigns  Ensuring regular supply of IFA Involvement of community leaders and influencers for motivating beneficiaries |
| **Strategies to improve compliance*** | Direct Observed Therapy by ASHA/AWW; family member, elders, community members) Creating habits and setting reminders help drive adherence Positive Beliefs Help Motivation Education and awareness about IFA treatments helps drive adherence Link life skill to IFA , Involve Teachers, Peer Students |
| **Strategies to Ensure Coverage of Complete Treatment of Anemic Cases till Recovery and Follow-Up with Prophylactic Doses** | Follow-up visits by ASHAs for checking compliance and ensuring repeat testing of Hb to assess improvement Implement electronic health records for tracking treatments Peer support groups for beneficiaries undergoing treatment Regular monitoring through AAM Counselling and education |
| **Training and orientation in AMB 2.0 intervention package, roles and responsibilities** | Organize workshops for healthcare providers Develop training manuals and resources Implement role-playing scenarios for practical understanding |
| **Strengthening health facilities** | Assess facility readiness and resource availability Upgrade equipment and supply chains for anemia treatment Establish referral protocols between facilities |
| **Strengthening ICDS role in AMB** | Integrate anemia screening in ICDS programs Train ICDS workers on anemia education and referral processes Foster partnerships between health and ICDS for coordinated efforts Monitoring and supervision for health and ICDS workers |
| **Improvement in processes, records and documentation** | Standardize record-keeping procedures Conduct regular audits of patient records |
| **Creating awareness and demand generation** | Launch community health campaigns on anemia prevention Distribute educational materials in local languages Use social media to engage and inform communities |
| **Organize and strengthen quality monitoring systems** | Develop key performance indicators for monitoring Establish feedback loops for continuous improvement Conduct regular quality assessments and staff evaluations |
| **Identify/create and prepare champions at various levels** | Select community leaders as champions for anemia advocacy Facilitate platforms for champions to share experiences |
| **Increase health systems accountability and community ownership** | Integrate Implementation research outcomes in Health Management Information System (HMIS) for periodic review SBCC, sharing of positive experiences Involve community representatives/leaders, administration |

*Compliance has been identified as a significant hurdle in anemia management. To improve and ensure adherence to treatment strategies, the following innovative approaches will be explored and implemented:

- **Regular Follow-Up Visits:** ASHA workers will conduct household visits, particularly targeting individuals with moderate to severe anemia. Each beneficiary identified as moderately or severely anemic will receive at least one visit per month for counseling and to assess compliance over the past week. These visits may occur every fortnight for more vulnerable groups, such as pregnant women and children aged 6-59 months.
- **Development of IT Tools:** An app or IT tool will be created for monitoring of compliance. This could involve features such as QR code scanning or missed call options that automatically register intake by beneficiaries, streamlining the tracking process.
- **Directly Observed Treatment (DOT):** To further ensure adherence, directly observed treatment will be implemented for all beneficiaries during Village Health and Nutrition Days (VHND) and Urban Health and Nutrition Days (UHND).
