# Supplementary material for: Implementation research to develop and optimize delivery models for evidence-based anemia control interventions in India: Protocol for the precision-driven response for anemia control and sustainable health (PRAKASH) study
Source: PLoS One. 2026 Jun 18;21(6):e0351414. doi: 10.1371/journal.pone.0351414 (PMC13278398; doi:10.1371/journal.pone.0351414)
Supplement: S3 Table — (DOCX) [file pone.0351414.s003.docx]

Supplementary Table 3: Indicators to be collected by IST-From HMIS and other sources

| Indicator | Data Source | Frequency |
| --- | --- | --- |
| **Indicators to be collected from HMIS** |  |  |
| Percentage of pregnant women screened for Haemoglobin (Hb) 4 or more than 4 times for respective ANCs | HMIS | Monthly |
| Percentage of PW providedfullcourse180Iron Folic Acid(IFA)tablets to total ANC registration | HMIS | Monthly |
| Number of PW having Hb level<=7g/dl(Out of total tested cases) | HMIS | Monthly |
| Number of PW treated for severe anemia (Hb<=7g/dl) (Out of total tested cases) | HMIS | Monthly |
| **Indicators to be collected from other sources by Implementation Support Team** |  |  |
| Percentage of testing points/platforms with functional digital hemoglobinometers | Testing site records/observations/ report | Monthly |
| Percentage of stakeholders trained for the 6 AMB 2.0 strategies | Training records/logs | Quarterly |
| Percentage of facilities implementing standardized treatment protocols for anemia management | Observation | Quarterly |
| Percentage of health platforms having adequate stock for IFA supplements, anti-helminthic supply | Inventory for medicine | Monthly |
| Percentage of cadre of health workers for whom the tracking system was operationalized | Ascertained by IST based on observation of the tracking system | Quarterly |
| Percentage of PDS/ICDS/other welfare schemes/centres distributing fortified rice | Records and observations by Implementation Support Team | Quarterly |
| Percentage and type/s of SBCC activities conducted in the defined study area in the last 3 months | Records at facilities and other platforms; observations of SBCC activities | Quarterly |
| Percentage of facilities/platforms displaying materials and/or using IEC materials during the SBCC activities/during counselling beneficiaries, at VHNDs, etc. in last 3 months | Records at facilities and other platforms; observations of SBCC activities | Quarterly |
| Percentage of beneficiaries screened for anemia at least once in last one year | ASHA records/health facility records | Monthly |
| % of beneficiaries who received the treatment for anemia | ASHA records/health facility records | Monthly |
| % of anemic repeat Hb done after 3 months | ASHA records/health facility records | Monthly |
| % of severe anaemic children referred for severe anemia (Hb<7 g/dl) | ASHA records/health facility records | Monthly |
| % of anemic individual who were referred to a specialist, received a consultation & further treatment | ASHA records/health facility records | Monthly |
| % birth attendants who report delayed cord clamping | ANC record/registers/Health facility records | Monthly |
| % patients diagnosed with acute/ chronic infections like malaria/HIV/Tb etc tested for anemia | Health facility records | Monthly |
| % of beneficiaries receiving albendazole tablet | Health facility records/Treatment logs/ASHA records | Biannual |
| % of beneficiaries given the full dose of Prophylactic IFA | ANC record/registers/Health facility records | Monthly |
