# Supplementary material for: Implementation research to develop and optimize delivery models for evidence-based anemia control interventions in India: Protocol for the precision-driven response for anemia control and sustainable health (PRAKASH) study
Source: PLoS One. 2026 Jun 18;21(6):e0351414. doi: 10.1371/journal.pone.0351414 (PMC13278398; doi:10.1371/journal.pone.0351414)
Supplement: Attachment — Submitted filename: Response to Reviewers.docx [file pone.0351414.s005.docx]

**PONE-D-25-62936:** Implementation Research to Develop and Optimize Delivery Models for Evidence-Based Anemia Control Interventions in India: Protocol for the Precision driven Response for Anemia Control and Sustainable Health (PRAKASH) Study

Dear Dr Jain,

Academic Editor
PLOS One

We sincerely thank you and the reviewers for the valuable comments and suggestions on our manuscript. We truly appreciate the time and effort invested in evaluating our work.

We are pleased to inform you that we have carefully addressed all the queries and comments raised. A detailed, point-wise response to each comment has been prepared and attached along with the revised manuscript.

We hope that the revisions meet your expectations. Please let us know if any further modifications or clarifications are required.

We look forward to your positive response and hope for an early decision.

Thank you for your consideration.

Kind Regards,

Journal Requirements:

When submitting your revision, we need you to address these additional requirements.

1. Please ensure that your manuscript meets PLOS ONE's style requirements, including those for file naming. The PLOS ONE style templates can be found at
[https://journals.plos.org/plosone/s/file?id=wjVg/PLOSOne_formatting_sample_main_body.pdf](https://track.editorialmanager.com/CL0/https:%2F%2Fjournals.plos.org%2Fplosone%2Fs%2Ffile%3Fid=wjVg%2FPLOSOne_formatting_sample_main_body.pdf/1/010f019cfea423e5-f64b5235-37ea-428b-bb95-c0a5bf08846f-000000/Nvr3FtyFaGihkmSTAaMB_xFS9vbc-2KYOM2bdhmseLQ=254) and [https://journals.plos.org/plosone/s/file?id=ba62/PLOSOne_formatting_sample_title_authors_affiliations.pdf](https://track.editorialmanager.com/CL0/https:%2F%2Fjournals.plos.org%2Fplosone%2Fs%2Ffile%3Fid=ba62%2FPLOSOne_formatting_sample_title_authors_affiliations.pdf/1/010f019cfea423e5-f64b5235-37ea-428b-bb95-c0a5bf08846f-000000/Wq4o8BiZozCKPuD-s9S1E0WVAp9_hOJcVJGCWOXvE9A=254)

**Response:** Thank you for the update. We have revised and updated the files in accordance with the template provided in the shared link. Please let us know if any further modifications are required.

2. PLOS requires an ORCID iD for the corresponding author in Editorial Manager on papers submitted after December 6th, 2016. Please ensure that you have an ORCID iD and that it is validated in Editorial Manager. To do this, go to ‘Update my Information’ (in the upper left-hand corner of the main menu), and click on the Fetch/Validate link next to the ORCID field. This will take you to the ORCID site and allow you to create a new iD or authenticate a pre-existing iD in Editorial Manager.

**Response:** We would like to inform you that we have completed the required task regarding the ORCID iD for the corresponding author. The ORCID iD has now been successfully created/authenticated and validated in the Editorial Manager system in accordance with PLOS submission requirements.

3. Please include your full ethics statement in the ‘Methods’ section of your manuscript file. In your statement, please include the full name of the IRB or ethics committee who approved or waived your study, as well as whether or not you obtained informed written or verbal consent. If consent was waived for your study, please include this information in your statement as well.

**Response:** Thank you for your constructive comment. The full name of the Institutional Review Board (IRB) and the ethics committee have been updated in the manuscript and is provided in the Ethics Approval section at line 212-216. Additionally, details regarding the consenting procedure are already present in the same section for clarity and completeness. We trust that these revisions adequately address the concern.

4. Your ethics statement should only appear in the Methods section of your manuscript. If your ethics statement is written in any section besides the Methods, please delete it from any other section.

**Response:** Thank you for your valuable suggestion regarding the placement of the ethics statement. We have carefully re-evaluated the manuscript in light of this comment. The ethics statement has been reviewed and is presented exclusively within the Methods section of the manuscript, with no duplication elsewhere.

We hope this revision meets the journal’s requirements and improves the clarity and organization of the manuscript.

5. One of the noted authors is a group or consortium [PRAKASH study group]. In addition to naming the author group, please list the individual authors and affiliations within this group in the acknowledgments section of your manuscript. Please also indicate clearly a lead author for this group along with a contact email address.

**Response:** The lead author for the group would be Dr Priyanka Gupta Bansal. The contact email id is [priyankaicmr@gmail.com](mailto:priyankaicmr@gmail.com). Also we have listed the individual authors and affiliations within this group in the acknowledgments section of your manuscript

6. Please provide a complete Data Availability Statement in the submission form, ensuring you include all necessary access information or a reason for why you are unable to make your data freely accessible. If your research concerns only data provided within your submission, please write "All data are in the manuscript and/or supporting information files" as your Data Availability Statement.

**Response:** Thank you for your comment. We would like to clarify that this manuscript is a protocol paper and, as such, does not include any empirical data. We hope this clarification addresses your concern. Please let us know if any further information is required.

7. Please amend either the title on the online submission form (via Edit Submission) or the title in the manuscript so that they are identical.

 Response: Thank you for your valuable suggestion. The title has been revised to ensure consistency, and it is now identical in both the online submission form and the manuscript. We appreciate your careful review and helpful feedback.

8. Please amend your list of authors on the manuscript to ensure that each author is linked to an affiliation. Authors’ affiliations should reflect the institution where the work was done (if authors moved subsequently, you can also list the new affiliation stating “current affiliation:….” as necessary).

**Response:** Thank you for your valuable suggestion. We have carefully considered your comment and ensure that the author’s affiliations are present in the manuscript accordingly. We appreciate your guidance in improving the quality of our work. Also we have listed the individual authors and affiliations ensure that each author is linked to an affiliation

9. One of the noted authors is a group or consortium [PRAKASH study group]. In addition to naming the author group, please list the individual authors and affiliations within this group in the acknowledgments section of your manuscript. Please also indicate clearly a lead author for this group along with a contact email address.

**Response:** The lead author for the group would be Dr Priyanka Gupta Bansal. The contact email id is [priyankaicmr@gmail.com](mailto:priyankaicmr@gmail.com). . Also we have listed the individual authors and affiliations within this group in the acknowledgments section of your manuscript

10. Please include a separate caption for each figure in your manuscript.

**Response:** We would like to kindly inform you that we have completed the requested revision regarding the figure captions in the manuscript. A separate and appropriate caption has now been provided for each figure, as per the journal’s requirements.

11. [Please include captions for your Supporting Information files at the end of your manuscript, and update any in-text citations to match accordingly. Please see our Supporting Information guidelines for more information: http://journals.plos.org/plosone/s/supporting-information.](http://track.editorialmanager.com/CL0/http:%2F%2Fjournals.plos.org%2Fplosone%2Fs%2Fsupporting-information./1/010f019cfea423e5-f64b5235-37ea-428b-bb95-c0a5bf08846f-000000/uvGslBwFlReEbgTu8-kAL2b3a10vDt5BllAY4v_TLGo=254)

**Response:** Thank you for your valuable suggestion. We have carefully considered your comment and have made the necessary revisions and included the supporting information at the end of the manuscript accordingly. We appreciate your guidance in improving the quality of our work.

12. If the reviewer comments include a recommendation to cite specific previously published works, please review and evaluate these publications to determine whether they are relevant and should be cited. There is no requirement to cite these works unless the editor has indicated otherwise. 

[Note: HTML markup is below. Please do not edit.]

Reviewers' comments:

Reviewer's Responses to Questions

**Comments to the Author**

1. Does the manuscript provide a valid rationale for the proposed study, with clearly identified and justified research questions?

The research question outlined is expected to address a valid academic problem or topic and contribute to the base of knowledge in the field.

Reviewer #1: Yes

**Response:** Thank you for your valuable inputs and for your kind appreciation of our work. We sincerely appreciate the time and effort you have taken to review the quality of our manuscript. Your comments have been very helpful in improving the quality and clarity of our manuscript.

2. Is the protocol technically sound and planned in a manner that will lead to a meaningful outcome and allow testing the stated hypotheses?

The manuscript should describe the methods in sufficient detail to prevent undisclosed flexibility in the experimental procedure or analysis pipeline, including sufficient outcome-neutral conditions (e.g. necessary controls, absence of floor or ceiling effects) to test the proposed hypotheses and a statistical power analysis where applicable. As there may be aspects of the methodology and analysis which can only be refined once the work is undertaken, authors should outline potential assumptions and explicitly describe what aspects of the proposed analyses, if any, are exploratory.

Reviewer #1: Yes

**Response:** Thank you for your kind appreciation of our work. We sincerely appreciate the time and effort you have taken to review the quality of our manuscript. Your comments have been very helpful in improving the quality and clarity of our manuscript.

3. Is the methodology feasible and described in sufficient detail to allow the work to be replicable?


Descriptions of methods and materials in the protocol should be reported in sufficient detail for another researcher to reproduce all experiments and analyses. The protocol should describe the appropriate controls, sample size calculations, and replication needed to ensure that the data are robust and reproducible.

Reviewer #1: No

**Response:** We thank the reviewer for the positive assessment of the methodological description. We would like to clarify that the methodology has been described in sufficient detail to ensure reproducibility and transparency. Regarding the concern about the use of appropriate controls, we respectfully note that this study is an implementation research (IR) study. In this context, the use of traditional control groups is not applicable, as IR designs focus on real-world evaluation of interventions within routine practice settings rather than controlled experimental comparisons. We also confirm that sample size calculations have been provided. We appreciate the reviewer’s valuable feedback, which helps strengthen the clarity of the manuscript.

4. Have the authors described where all data underlying the findings will be made available when the study is complete?

The [PLOS Data policy](https://track.editorialmanager.com/CL0/https:%2F%2Fjournals.plos.org%2Fplosone%2Fs%2Fmaterials-and-software-sharing/1/010f019cfea423e5-f64b5235-37ea-428b-bb95-c0a5bf08846f-000000/eR_6snjwlfjnxzljMzpZhPSV2jW8JLo_XEXkOAWUu3I=254) requires authors to make all data underlying the findings described in their manuscript fully available without restriction, with rare exceptions, at the time of publication. The data should be provided as part of the manuscript or its supporting information, or deposited in a public repository. For example, in addition to summary statistics, the data points behind means, medians and variance measures should be available. If there are restrictions on publicly sharing data—e.g. participant privacy or use of data from a third party—those must be specified.

Reviewer #1: No

**Response:** Thank you for your feedback. We would like to clarify that this manuscript is a protocol paper and, as such, does not include any empirical data. We hope this clarification addresses your concern. Please let us know if any further information is required.

5. Is the manuscript presented in an intelligible fashion and written in standard English?

PLOS ONE does not copyedit accepted manuscripts, so the language in submitted articles must be clear, correct, and unambiguous. Any typographical or grammatical errors should be corrected at revision, so please note any specific errors here.

Reviewer #1: No

**Response:** Thank you for your valuable suggestion. We have carefully revised the manuscript to improve its clarity and have presented the content in a more coherent and intelligible manner, adhering to standard academic writing conventions. We hope these revisions satisfactorily address your concern.

6. Review Comments to the Author

Please use the space provided to explain your answers to the questions above and, if applicable, provide comments about issues authors must address before this protocol can be accepted for publication. You may also include additional comments for the author, including concerns about research or publication ethics.

You may also provide optional suggestions and comments to authors that they might find helpful in planning their study.


(Please upload your review as an attachment if it exceeds 20,000 characters)

Reviewer #1: 1. Grammatical errors need to be addressed

**Response:** Thank you for your valuable suggestion. We have carefully revised the manuscript to address the grammatical errors adhering to standard academic writing conventions. We hope these revisions satisfactorily address your concern.

2. Too long sentence such as “To address these challenges, the Indian Council of Medical Research (ICMR) initiated the Precision driven Response for Anemia Control and Sustainable Health (PRAKASH) study—an implementation research initiative aimed at co-developing and optimizing a district-level model of evidence-based anemia control interventions”

**Response:** Thank you for your helpful suggestion. The sentence has been revised and shortened at line number 35-38 as recommended to improve clarity and readability.

3. Six pillars not clearly separated, use coon or numbers

**Response:** Thank you for your valuable suggestion. The six pillars have now been clearly separated and are presented using appropriate numbering (at line number 44-46) for improved clarity and organization. We hope this revision adequately addresses your concern.

4. Instead of Quantitative and qualitative methods; say Mixed-methods approaches will be used

**Response:** The terminology has been revised, and the study design now reflects the use of mixed-methods approaches (at line number 48) instead of separately referring to quantitative and qualitative methods. Thank you for your valuable suggestion.

5. Outcome statement is confusing, please rephrase

**Response:** The outcome statement has been revised at line number 53-54. We have carefully considered your comments, and we sincerely appreciate your guidance in improving the manuscript. Thank you once again for your time and support.

6. Conclusion missing in the abstract

**Response:** The abstract has been revised and the conclusion has now been included as recommended. Thank you for your valuable suggestion.

7. Discussion is repetition of aim and outcome, change it

**Response:** We thank the reviewer for this important observation. We acknowledge the concern that the Discussion section may appear to reiterate the study aim and key outcomes. In the revised manuscript, we have substantially restructured the Discussion to avoid repetition.

8. The abstract mentions AMB gaps but does not specify what evidence gap the study addresses.

**Response:** We thank the reviewer for this helpful comment. We agree that the original abstract did not clearly specify the exact evidence gap addressed by the study. This has now been revised. The abstract has been updated at line number 35-36 to explicitly state the evidence gap that the study addresses, thereby improving clarity regarding the rationale and significance of the work

9. Outcome definitions unclear. “Impact, implementation and coverage indicators” are vague.

**Response:** We thank the reviewer for this valuable comment. We acknowledge the concern regarding the clarity of outcome definitions, particularly the use of the terms “impact, implementation, and coverage indicators.” This has now been addressed in the revised manuscript by clearly specifying the utility of the indicators. The changes are incorporated at line number 53-54

10. Keywords: In appropriate use of capital letters

**Response:** Thank you for your valuable suggestion. The keywords section has been revised, and the inappropriate use of capital letters has been corrected to ensure proper formatting. The changes are incorporated at line number 59,60.

11. A series of total five cross sectional surveys; please clarify why 5? A series of total five cross sectional surveys (baseline, mid line and end-line) only 3 timepoints mentioned? Secondly, at other place it says Two baseline surveys will be conducted at months 3 and 9? This is very confusing. Please clarify.

**Response:** The needful rectification has been done under the sections study design and setting and study phases at line numbers 140-141 and 410,411, 422 to 424, respectively.

12. Table 1 do not have prevalence for age 5 to 9 years?

**Response:** We thank the reviewer for this observation regarding Table 1. We would like to clarify that the age group 5–9 years has not been included for prevalence estimates because the nationally representative surveys, including NFHS, do not capture or report prevalence data for this specific age band. As a result, comparable population-level estimates for this age group are not available for inclusion. Accordingly, prevalence has not been reported for 5–9 years in order to maintain consistency with the available national survey data and to avoid presenting non-comparable estimates.

13. In sample size calculation, population groups are not consistent what is mentioned in the objectives. Please ensure consistent population groups throughout the manuscript

**Response:** We thank the reviewer for this important observation regarding consistency of population groups across the objectives and the sample size calculation. We would like to clarify that the Anemia Mukt Bharat programme includes six beneficiary groups. These include children aged 6–59 months and 5-9 years, adolescent girls and boys, women of reproductive age, and pregnant women. Our focus is on all the groups; however, the four groups (children 6-59 months, adolescent girls (10-19 years), women of reproductive age (20-49 years), pregnant women) are selected for the impact indicators based on the existing group-specific prevalence of anemia. For, the remaining two groups (children 5-9 y and adolescent boys), our focus is on the process/coverage indicators. Also. we have updated for sample size calculations for children 5-9 years and adolescent boys. The changes are incorporated in the section sampling strategy.

We have ensured that this clarification has been consistently reflected throughout the revised manuscript to maintain alignment between the objectives, methodology, and sample size estimation. We appreciate the reviewer’s careful reading, which has helped improve the clarity and internal consistency of the manuscript.

14. In introduction mention why AMB has failed operationally.

**Response:** We thank the reviewer for this valuable suggestion. We would like to clarify that the operational challenges and reasons for implementation gaps in the Anemia Mukt Bharat (AMB) strategy have already been addressed in the Introduction, specifically in the third paragraph at line number 92-96. We appreciate the reviewer’s careful reading and constructive feedback, which has helped improve the clarity and structure of the manuscript.

15. The introduction does not clearly end with study objectives or hypotheses

**Response:** We would like to clarify that the study objectives have been explicitly stated in the final paragraph of the Introduction section. However, to improve clarity and better align with your suggestion, we have revised the text to more clearly highlight the objectives (and hypotheses, where applicable) at the end of the Introduction. We appreciate your feedback, which has helped us strengthen the presentation of our study.

16. Limitations not mentioned in the discussion

**Response:** Thank you for this thoughtful comment. As this manuscript presents a study protocol, the investigation is yet to be conducted, and, at this stage, we do not anticipate specific limitations beyond those inherent to the proposed design. However, we acknowledge that unforeseen challenges may arise during the implementation phase. We shall closely monitor the study’s progress and will address and transparently report any limitations encountered, along with appropriate mitigation strategies, in subsequent publications arising from this work.

17. Scalability strategy unclear.

**Response:** We thank the reviewer for this important comment. We acknowledge that the scalability strategy was not clearly articulated in the original manuscript. This has now been addressed in the revised version. We believe this clarification strengthens the discussion on scalability and enhances the policy relevance of the study. We appreciate the reviewer’s valuable input in improving the manuscript. The manuscript has been updated in the second paragraph of the discussion at line number 580-587.

**18.** The terminologies used to describe the sic pillars of interventions are not consistently used throughout the manuscript

**Response:** We have carefully reviewed the manuscript and ensured that the terminologies used to describe the six pillars of interventions are now applied consistently throughout the text. We believe this revision has improved the clarity and coherence of the manuscript. We appreciate your helpful suggestion.

19. In study phases it says “The recruitment of the participants will be done during the cross-sectional surveys which will be done during phase II and III of the study and will range from 15th December 2025 to 15 April 2029. Suggest to avoid using exact dates.

**Response:** Thank you for highlighting this point. We have revised the manuscript to remove the specific dates related to participant recruitment. The text now indicates that recruitment will occur during the cross-sectional surveys conducted in Phases II and III of the study, without specifying fixed calendar dates, to allow for necessary flexibility in the study timeline.

20. Objectives and outcomes are not aligning with each other

**Response:** We thank the reviewer for this important observation regarding the alignment between the study objectives and outcome indicators. We have carefully revisited the objectives and corresponding outcomes and would like to clarify and strengthen this alignment as follows:

First, the primary objective of the study is to reduce anemia prevalence to ≤20% among all target beneficiary groups within the study period. This is directly aligned with our primary outcome indicator, namely the prevalence of anemia defined using hemoglobin thresholds across population groups. Thus, the primary outcome is an explicit measure of the stated impact objective.

Second, the objective of achieving ≥80% effective population-based coverage of Anemia Mukt Bharat 2.0 interventions is captured through a comprehensive set of implementation and coverage indicators. These include testing, treatment initiation, adherence to iron and folic acid supplementation, follow-up testing, and consumption of fortified foods. Together, these indicators reflect both service delivery and utilization, thereby operationalizing the concept of “effective coverage.”

Third, the objective of co-designing and optimizing implementation strategies is inherently process-oriented. We acknowledge that this was not sufficiently reflected in the original outcome framework. In response, we have now incorporated additional process and implementation fidelity indicators (e.g., adherence to protocols, iterative adaptations, and documentation of implementation processes) to better capture this objective at page 15 line number 500-502.

Fourth, for the objective related to behavior change (Jan Andolan), we recognise that measuring only participation in community discussions may not fully capture behavioral outcomes. Accordingly, we have strengthened this section by including indicators on dietary practices (e.g., consumption frequency of iron-rich foods) and awareness levels to better reflect behavior change outcomes at pg 15 line number 479-482.

We sincerely appreciate the reviewer’s feedback, which has helped us strengthen the manuscript.

21. Division of village into four parts. This method is not standard probability sampling.

**Response:** Thank you for your observation regarding the division of the village into four parts and its classification as a non-probability sampling method. Upon careful consideration, we acknowledge that this approach does not constitute standard probability sampling. Therefore, we have revised our description to more accurately reflect the approach used. Relevant sections have been updated to ensure conceptual clarity and methodological accuracy. We appreciate your insightful feedback, which has helped us improve the rigor and transparency of our study. The necessary amendments are done under section sampling strategy.

22. Systematic sampling will be used to select the households, clarify how this will be done.

**Response:** Thank you for your valuable comment regarding the sampling strategy. Upon careful consideration, we have revised the description to more accurately reflect the approach used in our study. While systematic sampling was initially mentioned, we recognize that the actual method aligns more closely with multi stage cluster sampling. We have updated the relevant sections to ensure greater conceptual clarity and methodological accuracy. We appreciate your insightful feedback, which has helped us enhance the rigor and transparency of our study. The necessary amendments are done under section sampling strategy.

23. In data analysis section statistical methods, cluster adjustment, trend analysis, implementation outcomes analysis.

**Response:** Thank you for your valuable suggestion. We have updated the manuscript accordingly by incorporating the recommended statistical methods, including cluster adjustment, trend analysis, and implementation outcomes analysis. These revisions have been included under section data analysis in the paragraph on quantitative data section line number 519-534.

We appreciate your insightful feedback, which has helped us improve the rigor and clarity of our study.

24. In discussion add expected policy implications and generalizability to other LMICs.

**Response:** We thank the reviewer for this valuable suggestion. The discussion section has been revised accordingly to incorporate the expected policy implications of the study, as well as to highlight the potential generalizability of the findings to other low- and middle-income countries (LMICs). The necessary amendments have been incorporated in line number 600-603

25. Dissemination strategy is too generic, try to improve it more focused and clearer based on the objective.

**Response:** Thank you for your valuable feedback regarding the dissemination strategy. We appreciate your observation that the initial description was too generic. In response, we have substantially revised this section to ensure it is more focused, specific, and clearly aligned with the study’s objectives. The necessary amendments are incorporated in under sub section dissemination strategy line number 545-560.

7. PLOS authors have the option to publish the peer review history of their article ([what does this mean?](https://track.editorialmanager.com/CL0/https:%2F%2Fjournals.plos.org%2Fplosone%2Fs%2Feditorial-and-peer-review-process%23loc-peer-review-history/1/010f019cfea423e5-f64b5235-37ea-428b-bb95-c0a5bf08846f-000000/ByxjDQAgyOTn0QYOvK8WT6Zt5is0SGPALC6hKo8TKCE=254)). If published, this will include your full peer review and any attached files.


If you choose “no”, your identity will remain anonymous but your review may still be made public.


**Do you want your identity to be public for this peer review?** For information about this choice, including consent withdrawal, please see our [Privacy Policy](https://track.editorialmanager.com/CL0/https:%2F%2Fwww.plos.org%2Fprivacy-policy/1/010f019cfea423e5-f64b5235-37ea-428b-bb95-c0a5bf08846f-000000/Uwr2v6cgt1gT6erd5M9glkCO5hkvNwFYGqMEGsqTtTE=254).

Reviewer #1: No

Response: Thank you for providing this option. I would prefer not to have my identity disclosed publicly for this peer review. I understand and am comfortable with my review comments being published in an anonymous form.

[NOTE: If reviewer comments were submitted as an attachment file, they will be attached to this email and accessible via the submission site. Please log into your account, locate the manuscript record, and check for the action link "View Attachments". If this link does not appear, there are no attachment files.]

To ensure your figures meet our technical requirements, please review our figure guidelines: <https://journals.plos.org/plosone/s/figures>

You may also use PLOS’s free figure tool, NAAS, to help you prepare publication quality figures: <https://journals.plos.org/plosone/s/figures#loc-tools-for-figure-preparation>.

NAAS will assess whether your figures meet our technical requirements by comparing each figure against our figure specifications.

*In compliance with data protection regulations, you may request that we remove your personal registration details at any time.* [*(Remove my information/details)*](https://track.editorialmanager.com/CL0/https:%2F%2Fwww.editorialmanager.com%2Fpone%2Flogin.asp%3Fa=r/1/010f019cfea423e5-f64b5235-37ea-428b-bb95-c0a5bf08846f-000000/uqiblZnnDvtJm6jTt3iyB7SgsPq1VGt8PhR5Gwvh95k=254)*. Please contact the publication office if you have any questions.*
