# Supplementary material for: Implementation research to develop and optimize delivery models for evidence-based anemia control interventions in India: Protocol for the precision-driven response for anemia control and sustainable health (PRAKASH) study
Source: PLoS One. 2026 Jun 18;21(6):e0351414. doi: 10.1371/journal.pone.0351414 (PMC13278398; doi:10.1371/journal.pone.0351414)
Supplement: Peer review history

# Peer review: Decision Letter 0

**DOI:** 10.1371/journal.pone.0351414.r001

**Authors:** Yogesh Jain

17 Mar 2026

PONE-D-25-62936

Implementation Research to Develop and Optimize Delivery Models for Evidence-Based Anemia Control Interventions in India: Protocol for the Precision driven Response for Anemia Control and Sustainable Health (PRAKASH) Study

PLOS One

Dear Dr. Bansal,

Thank you for submitting your manuscript to PLOS ONE. After careful consideration, we feel that it has merit but does not fully meet PLOS ONE’s publication criteria as it currently stands. Therefore, we invite you to submit a revised version of the manuscript that addresses the points raised during the review process.

==============================

The manuscript has received an overall positive feedback, however, please make the below mentioned suggested edits to improve the quality of the manuscript.

==============================

Please submit your revised manuscript by May 01 2026 11:59PM. If you will need more time than this to complete your revisions, please reply to this message or contact the journal office at plosone@plos.org. When you're ready to submit your revision, log on to https://www.editorialmanager.com/pone/ and select the 'Submissions Needing Revision' folder to locate your manuscript file.

Please include the following items when submitting your revised manuscript:

- A letter that responds to each point raised by the academic editor and reviewer(s). You should upload this letter as a separate file labeled 'Response to Reviewers'.
- A marked-up copy of your manuscript that highlights changes made to the original version. You should upload this as a separate file labeled 'Revised Manuscript with Track Changes'.
- An unmarked version of your revised paper without tracked changes. You should upload this as a separate file labeled 'Manuscript'.

If you would like to make changes to your financial disclosure, please include your updated statement in your cover letter. Guidelines for resubmitting your figure files are available below the reviewer comments at the end of this letter.

If applicable, we recommend that you deposit your laboratory protocols in protocols.io to enhance the reproducibility of your results. Protocols.io assigns your protocol its own identifier (DOI) so that it can be cited independently in the future. For instructions see: https://journals.plos.org/plosone/s/submission-guidelines#loc-laboratory-protocols. Additionally, PLOS ONE offers an option for publishing peer-reviewed Lab Protocol articles, which describe protocols hosted on protocols.io. Read more information on sharing protocols at https://plos.org/protocols?utm_medium=editorial-email&utm_source=authorletters&utm_campaign=protocols.

We look forward to receiving your revised manuscript.

Kind regards,

Yogesh Kumar Jain, PhD

Academic Editor

PLOS One

Journal Requirements:

When submitting your revision, we need you to address these additional requirements.

1. Please ensure that your manuscript meets PLOS ONE's style requirements, including those for file naming. The PLOS ONE style templates can be found at

https://journals.plos.org/plosone/s/file?id=wjVg/PLOSOne_formatting_sample_main_body.pdf and https://journals.plos.org/plosone/s/file?id=ba62/PLOSOne_formatting_sample_title_authors_affiliations.pdf

2. PLOS requires an ORCID iD for the corresponding author in Editorial Manager on papers submitted after December 6th, 2016. Please ensure that you have an ORCID iD and that it is validated in Editorial Manager. To do this, go to ‘Update my Information’ (in the upper left-hand corner of the main menu), and click on the Fetch/Validate link next to the ORCID field. This will take you to the ORCID site and allow you to create a new iD or authenticate a pre-existing iD in Editorial Manager.

3. Please include your full ethics statement in the ‘Methods’ section of your manuscript file. In your statement, please include the full name of the IRB or ethics committee who approved or waived your study, as well as whether or not you obtained informed written or verbal consent. If consent was waived for your study, please include this information in your statement as well.

4. Your ethics statement should only appear in the Methods section of your manuscript. If your ethics statement is written in any section besides the Methods, please delete it from any other section.

5. One of the noted authors is a group or consortium [PRAKASH study group]. In addition to naming the author group, please list the individual authors and affiliations within this group in the acknowledgments section of your manuscript. Please also indicate clearly a lead author for this group along with a contact email address.

6. Please provide a complete Data Availability Statement in the submission form, ensuring you include all necessary access information or a reason for why you are unable to make your data freely accessible. If your research concerns only data provided within your submission, please write "All data are in the manuscript and/or supporting information files" as your Data Availability Statement.

7. Please amend either the title on the online submission form (via Edit Submission) or the title in the manuscript so that they are identical.

8. Please amend your list of authors on the manuscript to ensure that each author is linked to an affiliation. Authors’ affiliations should reflect the institution where the work was done (if authors moved subsequently, you can also list the new affiliation stating “current affiliation:….” as necessary).

9. One of the noted authors is a group or consortium [PRAKASH study group]. In addition to naming the author group, please list the individual authors and affiliations within this group in the acknowledgments section of your manuscript. Please also indicate clearly a lead author for this group along with a contact email address.

10. Please include a separate caption for each figure in your manuscript.

11. Please include captions for your Supporting Information files at the end of your manuscript, and update any in-text citations to match accordingly. Please see our Supporting Information guidelines for more information: http://journals.plos.org/plosone/s/supporting-information.

12. If the reviewer comments include a recommendation to cite specific previously published works, please review and evaluate these publications to determine whether they are relevant and should be cited. There is no requirement to cite these works unless the editor has indicated otherwise.

[Note: HTML markup is below. Please do not edit.]

Reviewers' comments:

Reviewer's Responses to Questions

**Comments to the Author**

1. Does the manuscript provide a valid rationale for the proposed study, with clearly identified and justified research questions?

The research question outlined is expected to address a valid academic problem or topic and contribute to the base of knowledge in the field.

Reviewer #1: Yes

**********

2. Is the protocol technically sound and planned in a manner that will lead to a meaningful outcome and allow testing the stated hypotheses?

The manuscript should describe the methods in sufficient detail to prevent undisclosed flexibility in the experimental procedure or analysis pipeline, including sufficient outcome-neutral conditions (e.g. necessary controls, absence of floor or ceiling effects) to test the proposed hypotheses and a statistical power analysis where applicable. As there may be aspects of the methodology and analysis which can only be refined once the work is undertaken, authors should outline potential assumptions and explicitly describe what aspects of the proposed analyses, if any, are exploratory.

Reviewer #1: Yes

**********

3. Is the methodology feasible and described in sufficient detail to allow the work to be replicable?

Descriptions of methods and materials in the protocol should be reported in sufficient detail for another researcher to reproduce all experiments and analyses. The protocol should describe the appropriate controls, sample size calculations, and replication needed to ensure that the data are robust and reproducible.

Reviewer #1: No

**********

4. Have the authors described where all data underlying the findings will be made available when the study is complete?

The PLOS Data policy requires authors to make all data underlying the findings described in their manuscript fully available without restriction, with rare exception, at the time of publication. The data should be provided as part of the manuscript or its supporting information, or deposited to a public repository. For example, in addition to summary statistics, the data points behind means, medians and variance measures should be available. If there are restrictions on publicly sharing data—e.g. participant privacy or use of data from a third party—those must be specified.

Reviewer #1: No

**********

5. Is the manuscript presented in an intelligible fashion and written in standard English?

PLOS ONE does not copyedit accepted manuscripts, so the language in submitted articles must be clear, correct, and unambiguous. Any typographical or grammatical errors should be corrected at revision, so please note any specific errors here.

Reviewer #1: No

**********

6. Review Comments to the Author

Please use the space provided to explain your answers to the questions above and, if applicable, provide comments about issues authors must address before this protocol can be accepted for publication. You may also include additional comments for the author, including concerns about research or publication ethics.

You may also provide optional suggestions and comments to authors that they might find helpful in planning their study.

(Please upload your review as an attachment if it exceeds 20,000 characters)

Reviewer #1: 1. Grammatical errors need to be addressed

2. Too long sentence such as “To address these challenges, the Indian Council of Medical Research (ICMR) initiated the Precision driven Response for Anemia Control and Sustainable Health (PRAKASH) study—an implementation research initiative aimed at co-developing and optimizing a district-level model of evidence-based anemia control interventions”

3. Six pillars not clearly separated, use coon or numbers

4. Instead of Quantitative and qualitative methods; say Mixed-methods approaches will be used

5. Outcome statement is confusing, please rephrase

6. Conclusion missing in the abstract

7. Discussion is repetition of aim and outcome, change it

8. The abstract mentions AMB gaps but does not specify what evidence gap the study addresses.

9. Outcome definitions unclear. “Impact, implementation and coverage indicators” are vague.

10. Keywords: In appropriate use of capital letters

11. A series of total five cross sectional surveys; please clarify why 5? A series of total five cross sectional surveys (baseline, mid line and end-line) only 3 timepoints mentioned? Secondly, at other place it says Two baseline surveys will be conducted at months 3 and 9? This is very confusing. Please clarify.

12. Table 1 do not have prevalence for age 5 to 9 years?

13. In sample size calculation, population groups are not consistent what is mentioned in the objectives. Please ensure consistent population groups throughout the manuscript

14. In introduction mention why AMB has failed operationally.

15. The introduction does not clearly end with study objectives or hypotheses

16. Limitations not mentioned in the discussion

17. Scalability strategy unclear.

18. The terminologies used to describe the sic pillars of interventions are not consistently used throughout the manuscript

19. In study phases it says “The recruitment of the participants will be done during the cross-sectional surveys which will be done during phase II and III of the study and will range from 15th December 2025 to 15 April 2029. Suggest to avoid using exact dates.

20. Objectives and outcomes are not aligning with each other

21. Division of village into four parts. This method is not standard probability sampling.

22. Systematic sampling will be used to select the households, clarify how this will be done.

23. In data analysis section statistical methods, cluster adjustment, trend analysis, implementation outcomes analysis.

24. In discussion add expected policy implications and generalizability to other LMICs.

25. Dissemination strategy is too generic, try to improve it more focused and clearer based on the objective.

**********

7. PLOS authors have the option to publish the peer review history of their article (what does this mean?). If published, this will include your full peer review and any attached files.

If you choose “no”, your identity will remain anonymous but your review may still be made public.

**Do you want your identity to be public for this peer review?**  For information about this choice, including consent withdrawal, please see our Privacy Policy.

Reviewer #1: No

**********

[NOTE: If reviewer comments were submitted as an attachment file, they will be attached to this email and accessible via the submission site. Please log into your account, locate the manuscript record, and check for the action link "View Attachments". If this link does not appear, there are no attachment files.]

To ensure your figures meet our technical requirements, please review our figure guidelines: https://journals.plos.org/plosone/s/figures

You may also use PLOS’s free figure tool, NAAS, to help you prepare publication quality figures: https://journals.plos.org/plosone/s/figures#loc-tools-for-figure-preparation.

NAAS will assess whether your figures meet our technical requirements by comparing each figure against our figure specifications.

# Author comment: Author response to Decision Letter 1

**DOI:** 10.1371/journal.pone.0351414.r002

23 Apr 2026

Reviewers' comments:

Reviewer's Responses to Questions

Comments to the Author

1. Does the manuscript provide a valid rationale for the proposed study, with clearly identified and justified research questions?

The research question outlined is expected to address a valid academic problem or topic and contribute to the base of knowledge in the field.

Reviewer #1: Yes

Response: Thank you for your valuable inputs and for your kind appreciation of our work. We sincerely appreciate the time and effort you have taken to review the quality of our manuscript. Your comments have been very helpful in improving the quality and clarity of our manuscript.

________________________________________

2. Is the protocol technically sound and planned in a manner that will lead to a meaningful outcome and allow testing the stated hypotheses?

The manuscript should describe the methods in sufficient detail to prevent undisclosed flexibility in the experimental procedure or analysis pipeline, including sufficient outcome-neutral conditions (e.g. necessary controls, absence of floor or ceiling effects) to test the proposed hypotheses and a statistical power analysis where applicable. As there may be aspects of the methodology and analysis which can only be refined once the work is undertaken, authors should outline potential assumptions and explicitly describe what aspects of the proposed analyses, if any, are exploratory.

Reviewer #1: Yes

Response: Thank you for your kind appreciation of our work. We sincerely appreciate the time and effort you have taken to review the quality of our manuscript. Your comments have been very helpful in improving the quality and clarity of our manuscript.

________________________________________

3. Is the methodology feasible and described in sufficient detail to allow the work to be replicable?

Descriptions of methods and materials in the protocol should be reported in sufficient detail for another researcher to reproduce all experiments and analyses. The protocol should describe the appropriate controls, sample size calculations, and replication needed to ensure that the data are robust and reproducible.

Reviewer #1: No

Response: We thank the reviewer for the positive assessment of the methodological description. We would like to clarify that the methodology has been described in sufficient detail to ensure reproducibility and transparency. Regarding the concern about the use of appropriate controls, we respectfully note that this study is an implementation research (IR) study. In this context, the use of traditional control groups is not applicable, as IR designs focus on real-world evaluation of interventions within routine practice settings rather than controlled experimental comparisons. We also confirm that sample size calculations have been provided. We appreciate the reviewer’s valuable feedback, which helps strengthen the clarity of the manuscript.________________________________________

4. Have the authors described where all data underlying the findings will be made available when the study is complete?

The PLOS Data policy requires authors to make all data underlying the findings described in their manuscript fully available without restriction, with rare exceptions, at the time of publication. The data should be provided as part of the manuscript or its supporting information, or deposited in a public repository. For example, in addition to summary statistics, the data points behind means, medians and variance measures should be available. If there are restrictions on publicly sharing data—e.g. participant privacy or use of data from a third party—those must be specified.

Reviewer #1: No

Response: Thank you for your feedback. We would like to clarify that this manuscript is a protocol paper and, as such, does not include any empirical data. We hope this clarification addresses your concern. Please let us know if any further information is required.

________________________________________

5. Is the manuscript presented in an intelligible fashion and written in standard English?

PLOS ONE does not copyedit accepted manuscripts, so the language in submitted articles must be clear, correct, and unambiguous. Any typographical or grammatical errors should be corrected at revision, so please note any specific errors here.

Reviewer #1: No

Response: Thank you for your valuable suggestion. We have carefully revised the manuscript to improve its clarity and have presented the content in a more coherent and intelligible manner, adhering to standard academic writing conventions. We hope these revisions satisfactorily address your concern.

________________________________________

6. Review Comments to the Author

Please use the space provided to explain your answers to the questions above and, if applicable, provide comments about issues authors must address before this protocol can be accepted for publication. You may also include additional comments for the author, including concerns about research or publication ethics.

You may also provide optional suggestions and comments to authors that they might find helpful in planning their study.

(Please upload your review as an attachment if it exceeds 20,000 characters)

Reviewer #1: 1. Grammatical errors need to be addressed

Response: Thank you for your valuable suggestion. We have carefully revised the manuscript to address the grammatical errors adhering to standard academic writing conventions. We hope these revisions satisfactorily address your concern.

2. Too long sentence such as “To address these challenges, the Indian Council of Medical Research (ICMR) initiated the Precision driven Response for Anemia Control and Sustainable Health (PRAKASH) study—an implementation research initiative aimed at co-developing and optimizing a district-level model of evidence-based anemia control interventions”

Response: Thank you for your helpful suggestion. The sentence has been revised and shortened at line number 35-38 as recommended to improve clarity and readability.

3. Six pillars not clearly separated, use coon or numbers

Response: Thank you for your valuable suggestion. The six pillars have now been clearly separated and are presented using appropriate numbering (at line number 44-46) for improved clarity and organization. We hope this revision adequately addresses your concern.

4. Instead of Quantitative and qualitative methods; say Mixed-methods approaches will be used

Response: The terminology has been revised, and the study design now reflects the use of mixed-methods approaches (at line number 48) instead of separately referring to quantitative and qualitative methods. Thank you for your valuable suggestion.

5. Outcome statement is confusing, please rephrase

Response: The outcome statement has been revised at line number 53-54. We have carefully considered your comments, and we sincerely appreciate your guidance in improving the manuscript. Thank you once again for your time and support.

6. Conclusion missing in the abstract

Response: The abstract has been revised and the conclusion has now been included as recommended. Thank you for your valuable suggestion.

7. Discussion is repetition of aim and outcome, change it

Response: We thank the reviewer for this important observation. We acknowledge the concern that the Discussion section may appear to reiterate the study aim and key outcomes. In the revised manuscript, we have substantially restructured the Discussion to avoid repetition.

8. The abstract mentions AMB gaps but does not specify what evidence gap the study addresses.

Response: We thank the reviewer for this helpful comment. We agree that the original abstract did not clearly specify the exact evidence gap addressed by the study. This has now been revised. The abstract has been updated at line number 35-36 to explicitly state the evidence gap that the study addresses, thereby improving clarity regarding the rationale and significance of the work

9. Outcome definitions unclear. “Impact, implementation and coverage indicators” are vague.

Response: We thank the reviewer for this valuable comment. We acknowledge the concern regarding the clarity of outcome definitions, particularly the use of the terms “impact, implementation, and coverage indicators.” This has now been addressed in the revised manuscript by clearly specifying the utility of the indicators. The changes are incorporated at line number 53-54

10. Keywords: In appropriate use of capital letters

Response: Thank you for your valuable suggestion. The keywords section has been revised, and the inappropriate use of capital letters has been corrected to ensure proper formatting. The changes are incorporated at line number 59,60.

11. A series of total five cross sectional surveys; please clarify why 5? A series of total five cross sectional surveys (baseline, mid line and end-line) only 3 timepoints mentioned? Secondly, at other place it says Two baseline surveys will be conducted at months 3 and 9? This is very confusing. Please clarify.

Response: The needful rectification has been done under the sections study design and setting and study phases at line numbers 140-141 and 410,411, 422 to 424, respectively.

12. Table 1 do not have prevalence for age 5 to 9 years?

Response: We thank the reviewer for this observation regarding Table 1. We would like to clarify that the age group 5–9 years has not been included for prevalence estimates because the nationally representative surveys, including NFHS, do not capture or report prevalence data for this specific age band. As a result, comparable population-level estimates for this age group are not available for inclusion. Accordingly, prevalence has not been reported for 5–9 years in order to maintain consistency with the available national survey data and to avoid presenting non-comparable estimates.

13. In sample size calculation, population groups are not consistent what is mentioned in the objectives. Please ensure consistent population groups throughout the manuscript

Response: We thank the reviewer for this important observation regarding consistency of population groups across the objectives and the sample size calculation. We would like to clarify that the Anemia Mukt Bharat programme includes six beneficiary groups. These include children aged 6–59 months and 5-9 years, adolescent girls and boys, women of reproductive age, and pregnant women. Our focus is on all the groups; however, the four groups (children 6-59 months, adolescent girls (10-19 years), women of reproductive age (20-49 years), pregnant women) are selected for the impact indicators based on the existing group-specific prevalence of anemia. For, the remaining two groups (children 5-9 y and adolescent boys), our focus is on the process/coverage indicators. Also. we have updated for sample size calculations for children 5-9 years and adolescent boys. The changes are incorporated in the section sampling strategy.

We have ensured that this clarification has been consistently reflected throughout the revised manuscript to maintain alignment between the objectives, methodology, and sample size estimation. We appreciate the reviewer’s careful reading, which has helped improve the clarity and internal consistency of the manuscript.

14. In introduction mention why AMB has failed operationally.

Response: We thank the reviewer for this valuable suggestion. We would like to clarify that the operational challenges and reasons for implementation gaps in the Anemia Mukt Bharat (AMB) strategy have already been addressed in the Introduction, specifically in the third paragraph at line number 92-96. We appreciate the reviewer’s careful reading and constructive feedback, which has helped improve the clarity and structure of the manuscript.

15. The introduction does not clearly end with study objectives or hypotheses

Response: We would like to clarify that the study objectives have been explicitly stated in the final paragraph of the Introduction section. However, to improve clarity and better align with your suggestion, we have revised the text to more clearly highlight the objectives (and hypotheses, where applicable) at the end of the Introduction. We appreciate your feedback, which has helped us strengthen the presentation of our study.

16. Limitations not mentioned in the discussion

Response: Thank you for this thoughtful comment. As this manuscript presents a study protocol, the investigation is yet to be conducted, and, at this stage, we do not anticipate specific limitations beyond those inherent to the proposed design. However, we acknowledge that unforeseen challenges may arise during the implementation phase. We shall closely monitor the study’s progress and will address and transparently report any limitations encountered, along with appropriate mitigation strategies, in subsequent publications arising from this work.

17. Scalability strategy unclear.

Response: We thank the reviewer for this important comment. We acknowledge that the scalability strategy was not clearly articulated in the original manuscript. This has now been addressed in the revised version. We believe this clarification strengthens the discussion on scalability and enhances the policy relevance of the study. We appreciate the reviewer’s valuable input in improving the manuscript. The manuscript has been updated in the second paragraph of the discussion at line number 580-587.

18. The terminologies used to describe the sic pillars of interventions are not consistently used throughout the manuscript

Response: We have carefully reviewed the manuscript and ensured that the terminologies used to describe the six pillars of interventions are now applied consistently throughout the text. We believe this revision has improved the clarity and coherence of the manuscript. We appreciate your helpful suggestion.

19. In study phases it says “The recruitment of the participants will be done during the cross-sectional surveys which will be done during phase II and III of the study and will range from 15th December 2025 to 15 April 2029. Suggest to avoid using exact dates.

Response: Thank you for highlighting this point. We have revised the manuscript to remove the specific dates related to participant recruitment. The text now indicates that recruitment will occur during the cross-sectional surveys conducted in Phases II and III of the study, without specifying fixed calendar dates, to allow for necessary flexibility in the study timeline.

20. Objectives and outcomes are not aligning with each other

Response: We thank the reviewer for this important observation regarding the alignment between the study objectives and outcome indicators. We have carefully revisited the objectives and corresponding outcomes and would like to clarify and strengthen this alignment as follows:

First, the primary objective of the study is to reduce anemia prevalence to ≤20% among all target beneficiary groups within the study period. This is directly aligned with our primary outcome indicator, namely the prevalence of anemia defined using hemoglobin thresholds across population groups. Thus, the primary outcome is an explicit measure of the stated impact objective.

Second, the objective of achieving ≥80% effective population-based coverage of Anemia Mukt Bharat 2.0 interventions is captured through a comprehensive set of implementation and coverage indicators. These include testing, treatment initiation, adherence to iron and folic acid supplementation, follow-up testing, and consumption of fortified foods. Together, these indicators reflect both service delivery and utilization, thereby operationalizing the concept of “effective coverage.”

Third, the objective of co-designing and optimizing implementation strategies is inherently process-oriented. We acknowledge that this was not sufficiently reflected in the original outcome framework. In response, we have now incorporated additional process and implementation fidelity indicators (e.g., adherence to protocols, iterative adaptations, and documentation of implementation processes) to better capture this objective at page 15 line number 500-502.

Fourth, for the objective related to behavior change (Jan Andolan), we reco

Attachment Submitted filename: Response to Reviewers.docx

# Editor report: Decision Letter 1

**DOI:** 10.1371/journal.pone.0351414.r003

**Authors:** Yogesh Jain, Yogesh Jain

27 May 2026

Implementation research to develop and optimize delivery models for evidence-based anemia control interventions in India: protocol for the precision-driven response for anemia control and sustainable health (PRAKASH) study

PONE-D-25-62936R1

Dear Dr. Bansal,

We’re pleased to inform you that your manuscript has been judged scientifically suitable for publication and will be formally accepted for publication once it meets all outstanding technical requirements.

Within one week, you’ll receive an e-mail detailing the required amendments. When these have been addressed, you’ll receive a formal acceptance letter and your manuscript will be scheduled for publication.

An invoice will be generated when your article is formally accepted. Please note, if your institution has a publishing partnership with PLOS and your article meets the relevant criteria, all or part of your publication costs will be covered. Please make sure your user information is up-to-date by logging into Editorial Manager at Editorial Manager® and clicking the ‘Update My Information' link at the top of the page. For questions related to billing, please contact billing support.

If your institution or institutions have a press office, please notify them about your upcoming paper to help maximize its impact. If they’ll be preparing press materials, please inform our press team as soon as possible -- no later than 48 hours after receiving the formal acceptance. Your manuscript will remain under strict press embargo until 2 pm Eastern Time on the date of publication. For more information, please contact onepress@plos.org.

Kind regards,

Yogesh Kumar Jain, PhD

Academic Editor

PLOS One

Additional Editor Comments (optional):

Reviewers' comments:

# Editor report: Acceptance letter

**DOI:** 10.1371/journal.pone.0351414.r004

**Authors:** Yogesh Jain, Yogesh Jain

PONE-D-25-62936R1

PLOS One

Dear Dr. Bansal,

I'm pleased to inform you that your manuscript has been deemed suitable for publication in PLOS One. Congratulations! Your manuscript is now being handed over to our production team.

At this stage, our production department will prepare your paper for publication. This includes ensuring the following:

* All references, tables, and figures are properly cited

* All relevant supporting information is included in the manuscript submission,

* There are no issues that prevent the paper from being properly typeset

You will receive further instructions from the production team, including instructions on how to review your proof when it is ready. Please keep in mind that we are working through a large volume of accepted articles, so please give us a few days to review your paper and let you know the next and final steps.

Lastly, if your institution or institutions have a press office, please let them know about your upcoming paper now to help maximize its impact. If they'll be preparing press materials, please inform our press team within the next 48 hours. Your manuscript will remain under strict press embargo until 2 pm Eastern Time on the date of publication. For more information, please contact onepress@plos.org.

You will receive an invoice from PLOS for your publication fee after your manuscript has reached the completed accept phase. If you receive an email requesting payment before acceptance or for any other service, this may be a phishing scheme. Learn how to identify phishing emails and protect your accounts at https://explore.plos.org/phishing.

If we can help with anything else, please email us at customercare@plos.org.

Thank you for submitting your work to PLOS ONE and supporting open access.

Kind regards,

PLOS ONE Editorial Office Staff

on behalf of

Dr. Yogesh Kumar Jain

Academic Editor

PLOS One
